# Truncated hemoglobin 1 is a new player in *Chlamydomonas reinhardtii* acclimation to sulfur deprivation

**DOI:** 10.1371/journal.pone.0186851

**Published:** 2017-10-19

**Authors:** Ekaterina Minaeva, Zhanneta Zalutskaya, Valentina Filina, Elena Ermilova

**Affiliations:** Biological Faculty, Saint-Petersburg State University, Saint-Petersburg, Russia; Universidade de Lisboa Instituto Superior de Agronomia, PORTUGAL

## Abstract

Truncated hemoglobins constitute a large family, present in bacteria, in archaea and in eukaryotes. However, a majority of physiological functions of these proteins remains to be elucidated. Identification and characterization of a novel role of truncated hemoglobins in the model alga provides a framework for a more complete understanding of their biological functions. Here, we use quantitative RT-PCR to show that three truncated hemoglobins of *Chlamydomonas reinhardtii*, *THB1*, *THB2* and *THB12*, are induced under conditions of depleted sulfur (S) supply. THB1 underexpression results in the decrease in cell size, as well in levels of proteins, chlorophylls and mRNA of several S-responsive genes under S starvation. We provide evidence that knock-down of THB1 enhances NO production under S deprivation. In S-deprived cells, a subset of S limitation-responsive genes is controlled by NO in THB1-dependent pathway. Moreover, we demonstrate that deficiency for S represses the nitrate reduction and that THB1 is involved in this control. Thus, our data support the idea that in S-deprived cells THB1 plays a dual role in NO detoxification and in coordinating sulfate limitation with nitrate assimilation. This study uncovers a new function for the *Chlamydomonas reinhardtii* THB1 in the control of proper response to S deprivation.

## Introduction

Hemoglobins (Hbs) constitute a large superfamily of the globular heme-containing metalloproteins, present in archaea, in bacteria, and in eukaryotes. The phylogenetic complexity of Hbs is equaled by a wide diversification of reactions catalyzed by the hemoproteins [1]. In plants, there are now three main types of Hbs: symbiotic, non-symbiotic and truncated (TrHb) [2–4]. Symbiotic and non-symbiotic Hbs belong to the myoglobin-like family and have the canonical 3-on-3 α-helical fold (3/3 Hbs). The sequences of TrHb are 20–40 amino acid residues shorter than 3/3 Hbs [5]. In contrast to full-length Hbs, TrHbs tertiary structure share a characteristic helix arrangement folded in a 2-on-2 α-helical sandwich. Three groups of TrHbs, called TrHb1, TrHb2, and TrHb3, have been identified based on protein sequence analysis [5, 6]. The key structural features of TrHbs family have been summarized in previous reviews [7, 8]. Although the presence of TrHbs is widespread in bacteria and in eukaryotes [5, 9], their role has not yet been fully elucidated. It has been proposed that one particular function of these TrHbs might be ascribed to modulation of nitric oxide (NO) levels inside cells [10, 11]. However, only a few studies have addressed this issue. The search for TrHbs physiological functions in prokaryotes and eukaryotes is undergoing a surge of interest.

To study many fundamental problems in biology, the unicellular green alga *Chlamydomonas reinhardtii* has proved an excellent model organism [12, 13]. The genome of *C*. *reinhardtii* contains 12 genes encoding TrHbs (TrHb1 group) named as *THB1-12* [14, 15]. Little physiological information is available for these proteins. THB1 was recently shown to be linked to nitrogen metabolism [16] while THB8 was vital for anoxic growth [14]. Both of these THBs were proposed to participate in NO-dependent signaling pathways. Moreover, THB1 was capable of interacting with nitrate reductase (NR) to scavenge NO [17, 18]. Despite the proven importance of THB1 in the control of nitrate reduction, other roles of THBs are largely unknown. Further characterization of the pathways that might be controlled by THBs to coordinate nutrient stress responses is called for.

Sulfur (S) is one of the essential macroelements in plant nutrition [19]. Most plants assimilate S as a sulfate and transport it to the plastid, where primary S metabolism takes place. Because S can be limiting in the environment, many organisms, including photosynthetic *C*. *reinhardtii*, have evolved mechanisms to adjust to S deprivation conditions. Moreover, several lines of research highlighted the existence of crosstalks operating between sulfate and nitrate metabolism in photosynthetic eukaryotes [20, 21, 22, 23, 24]. Deficiency for one element was shown to repress the other pathway [25, 26, 27].

The adaptation of *C*. *reinhardtii* to S starvation has been well characterized recently [28, 29, 30]. During S limitation, *C*. *reinhardtii* cells demonstrate increased transcription of numerous genes encoding proteins associated with sulfate uptake and assimilation, internal S recycling and changes in metabolism [28]. However, the responses of *C*. *reinhardtii THB*1-12 genes to S limitation conditions have not been analyzed. This may serve to gain additional information on the function of these proteins. In the present work, we found that S deprivation induced transcription of *THB1*, *THB2* and *THB12*. Moreover, THB1 is needed for proper induction of some S limitation-responsive genes. Our data demonstrate that nitrate reduction is regulated by S starvation and that THB1 plays a role in this regulation.

## Materials and methods

### Strains and growth conditions

The strain cw15-325 (cw15*mt*^+^*arg7-8*) was kindly provided by Dr. M. Schroda (University of Kaiserslautern, Germany). Cells were grown mixotrophically in tris-acetate-phosphate (TAP) medium [http://www.chlamy.org/TAP.html, 29] under continuous illumination with white light at 22°C. The TAP medium was supplemented with 100 mg/l of arginine when required. To induce sulfur deprivation of the strains used, the cells grown in TAP medium in the light were washed twice with sulfur-free medium (TAP-S) and then were resuspended in TAP-S. S-free medium was prepared as reported previously [30]. At each harvesting times the number of cells was measured employing a counting chamber and the viable cells were estimated microscopically with use of 0.0125% (v/v) methylene blue (DIA-M, Russia) as described [31]. Stained (non-viable) and unstained (viable) cells were observed and counted. 400 cells from each sample were examined for three biological replicates. For size determination cells were imaged with a Leica TCS-SP5 confocal microscope (Leica-Microsystems, Germany) equipped with a HC PL APO 63× oil immersion. Excitation was performed with a 488-nm argon laser (30% power). Diameters of cells were determined using the software supplied by Intelligent Imaging Innovations. 300 cells were scored in each sample. The experiment was performed in triplicate.

### Gene expression

#### RNA isolation and cDNA synthesis

Total RNA was extracted as described previously [32] The RNA samples were treated with RNase-Free DNase I (Fermentas) to remove genomic DNA. The reaction was stopped with 0.43 μL of 50 mM EDTA at 80°C for 10 min. Subsequently, RNA concentration and purity (260/280 nm ratio) was determined using spectrophotometer (SmartSpec Plus, Bio-Rad). The verification of RNA integrity was carried out on 1.2% (w/v) denaturing agarose gel prepared in 1X TAE buffer (40 мM Tris, 20 мM acetate, 1 мM EDTA) at 100 V for 20 min. The electrophoresed samples were stained with ethidium bromide and visualized under UV light using gel documentation system (BioDoc-It^™^ Imaging system). RNA concentration was adjusted to 1 μg/μl, and cDNA strand was synthesized using RevertAid HMinus First Strand cDNA Synthesis Kit according to the manufacturer’s instructions (Thermo Scientific).

#### Real time quantitative RT-PCR

Real time quantitative RT-PCR (RT-qPCR) reactions were performed on the Light Cycler Instrument (CFX96 Real-Time PCR Detection System, Bio Rad) using SYBR Green I as a fluorescent dye. Each reaction contained the master mix, 5% DMSO, 200 nM of each primer, and cDNA corresponding to 10 ng input RNA in the reverse transcriptase reaction. The primers are listed in S1 Table. Three technical replicates were used for each gene/primer combination. Primers for RT-qPCRs were chosen based on ≥ 90% primer efficiency and on a single melt curve. Melt curve peaks for the genes are shown in Figures B, E and J in S1 File. The amplification charts for the target genes are shown in Figures C, F, H and K in S1 File. Gene expression ratios were normalized against *RACK1* (receptor of activated protein kinase C; Cre06.g278222, formerly termed CBLP) using the ΔC_T_ and ΔΔC_T_ methods [33, 34]. Sulfur starvation treatments had no effect on the accumulation of *RACK1* transcripts in *C*. *reinhardtii* cells (S1 File). The accuracy and reproducibility of the real time assay was determined from low variation in C_T_ values across replicates in Tables A, C, E and G in S1 File. Values were obtained from at least two biological replicates; each replicate was analyzed three times. Student’s *t*-tests were used for statistical comparisons. P-values of <0.05 were considered as significant.

### Generation of THB1 knock-down strains

We constructed vector pChlamiRNA2-THB1 as described [35]. The primers used for plasmid preparation were 5'-ctagtGTGCGCTTTTCAGTAAAAAGAtctcgctgatcggcaccatgggggtggtggtgatcagcgctaTCTTATTACTGAAAAGCGCACg-3' and 5´-ctagcGTGCGCTTTTCAGTAATAAGAtagcgctgatcaccaccacccccatggtgccgatcagcgagaTCTTTTTACTGAAAAGCGCACa 3'. This plasmid (pChlamiRNA2*THB1*) or the empty vector were transformed into the stain cw15-325 following the protocol described by Kindle [36]. TAP agar without arginine was used for selection. The strains were screened out by using a RT-qPCR to confirm the reduced abundance of *THB1*mRNA.

### Determination of chlorophyll and protein contents

Chlorophyll content was determined using ethanol extraction. 1 ml of culture was centrifuged and the pellet was resuspended in 1 ml ethanol to extract pigments. Cellular debris was pelleted by centrifugation and chlorophyll a and b levels were determined spectrophotometrically (SmartSpec Plus, BioRad) in the supernatant, by measuring optical absorbance at 645 and 663 nm. Calculations of total chlorophyll (μg/ml) were performed as previously described [13].

For protein isolation, cells (1–2 10^6^ cells/ml in 10 ml) were harvested by centrifugation, the supernatant was discarded and the pellet was resuspended in 0.1 M DTT and 0.1 M Na_2_CO_3_. Then, 0.66 vol of 5% SDS and 30% sucrose were added to all samples. The protein concentration was determined by staining with amido black, using BSA as a standard [37].

### Measurement of NO

*C*. *reinhardtii* cells (45 μg/ml chlorophyll) were deprived of S for 15 min and incubated in the presence of 20 μM (4-amino-5-methylamino-2’7’-difluorofluorescein diacetate) dye (DAF-FM DA, Sigma-Aldrich). After 15 min the cells were washed, resuspended in S-free medium and then incubated for an additional 30 min to allow complete de-esterification of the intracellular diacetates. When indicated 2-(N,N diethylamino)-diazenolate 2-oxide sodium salt (DEA-NONOate, Sigma-Aldrich) and the selective NO scavenger 2-phenyl-4,4,5,5-tetramethylimidazoline-1-oxyl 3-oxide (cPTIO, Sigma Aldrich) were added to the medium to a final concentration of 50 or 100 μM and 300 μM, respectively. When indicated 2-(N,N diethylamino)-diazenolate 2-oxide sodium salt (DEA-NONOate, Sigma-Aldrich) and the selective NO scavenger 2-phenyl-4,4,5,5-tetramethylimidazoline-1-oxyl 3-oxide (cPTIO, Sigma Aldrich) were added to the medium to a final concentration of 50 or 100 μM and 300 μM, respectively; then cells were washed in S-free medium and analyzed after additional 30 min, as described above. Intracellular production of NO was measured using a microplate reader CLARIOstar (BMG). Excitation and emission wavelengths were set at 483±14 and 530±30 nm, respectively. Fluorescence intensity was expressed as arbitrary units per chlorophyll cells 10^6^. Cell autofluorescence was subtracted from the total fluorescence obtained. Three technical replicates per condition were included on each plate and each experiment was performed three times independently.

### Confocal microscopy

For confocal microscopy cells were grown and treated as described above (measurement of NO). Cells were visualized with a Leica TCS-SP5 confocal microscope (Leica-Microsystems, Germany) equipped with a HC PL APO 63×oil immersion objective. Excitation was performed with a 488-nm argon laser. The signals arising from the DAF-FM DA were collected on the channel between 500 and 544 nm. Chlorophyll autofluorescence was monitored across a window of 600–680 nm. Images were collected and processed with the Leica confocal software LAS AF (Leica-Microsystems, Germany). The experiment was performed in triplicate.

### Enzymatic assay for nitrate reductase activity

*C*. *reinhardtii* cells were grown in TAP and induced in 4 mM KNO_3_ medium with or without sulfur for 3 h. NR activity was determined as described previously [38]. NR was assayed by measuring the formation of nitrite from added nitrate and NADH in an incubation mixture containing in 1 mL: 60 mM potassium phosphate (pH 7.5), 50 mM KNO_3_, 0.1 mM NADH_2_, and 0.1 ml sample with about 0.5 mg Chl. Before addition of the other chemicals, cells were lysed with 5% toluene. 1 min before starting the NR activity measurements, 1 mM of the electron acceptor ferricyanide 1% (w/v) was added to activate the enzyme. After incubation for 30 min at 30°C the reaction was stopped by boiling (1 min), and the mixture was cleared by centrifugation (27,000g). For the determination of nitrite the supernatant was mixed with 1 ml sulfanilamide in 2 N HCI and 0.2% (w/v) N-(l-naphthyl)ethylenediamine. The absorption of the resulting violet color was measured at 540 nm against a blank. Activity values were expressed as milliunits (mU), defined as the amount of enzyme catalyzing the transformation of 1 nmol of substrate per min, relative to the chlorophyll content, determined as described above.

## Results

### Expression of THBs under sulfur deprivation

**Fig 1:**
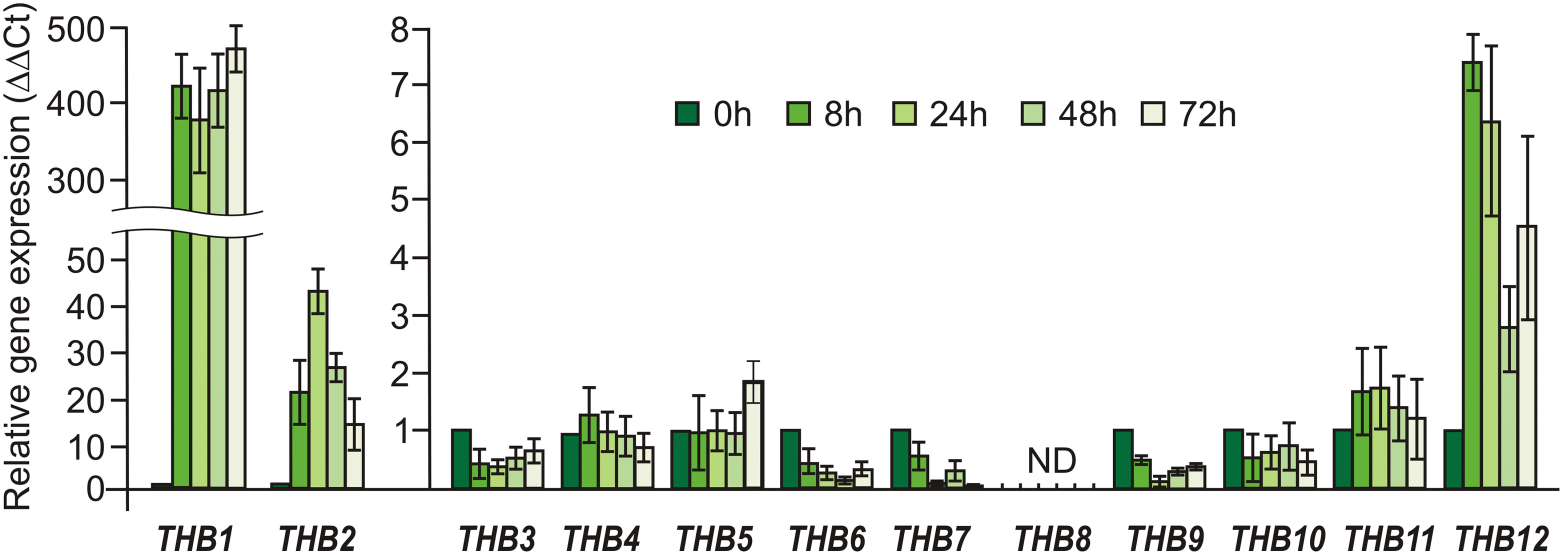
Effects of sulfur deprivation on the relative transcript abundance for genes encoding truncated hemoglobins THB1-12 in *Chlamydomonas reinhardtii*. Vegetative cells of cw15-325 were treated as described in Materials and methods. Levels of gene transcripts are given as times of relative abundance with respect to the housekeeping control gene (*RACK1*) that has a value of 1. Data are the means±SE from three biological and two technical replicates obtained by real-time RT-PCR. ND, transcripts are not detected. See Supporting experimental procedures (Figures A, B and C and Tables A and B in S1 File) for more information.

The *THB1* and *THB2* genes have been shown to be strongly regulated by nitrogen [18]. In order to examine whether the transcription of the *THB* genes are linked to the sulfur source, we monitored the expression patterns of *THB1-12* in S-free media in the presence of light (Fig 1).

The three genes, *THB1*, *THB2* and *THB12*, exhibited an increase of transcript level in S-starved cells in comparison to control cells. By contrast, four genes, *THB4*, *THB5*, *THB10* and *THB11*, did not show any differential expression under S deprivation conditions. The levels of the other four transcripts for *THB3*, *THB6*, *THB7* and *THB9* were even slightly reduced. Moreover, *THB8* was not detectable both in noninduced and in S-deprivation induced cells. THB1 was chosen for further analysis because it showed the highest expression levels in S-free medium.

**Fig 2:**
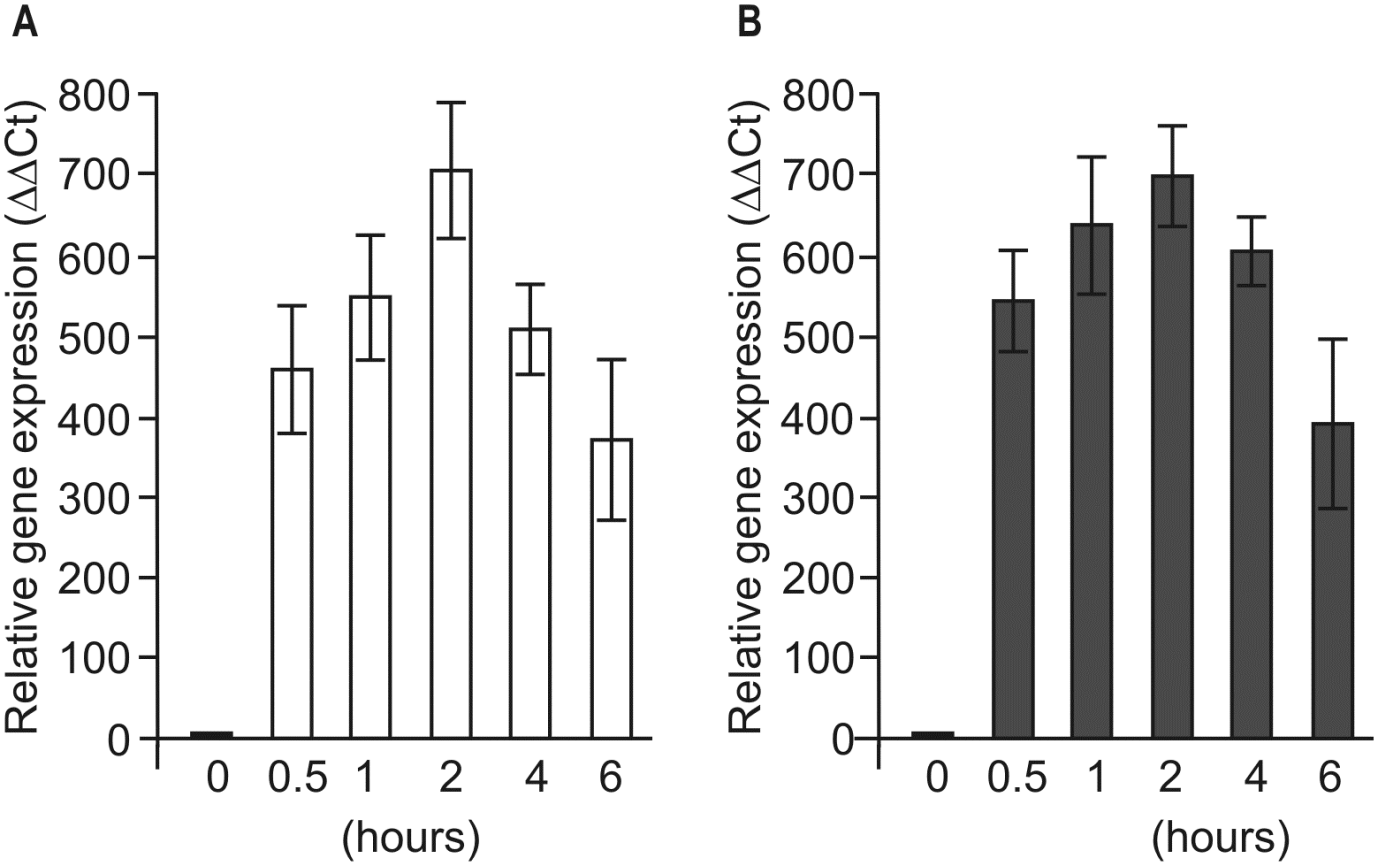
Effects of sulfur deprivation on *THB1* transcript accumulation under different light conditions. Light-grown *Chlamydomonas reinhardtii* cw15-325 cells were transferred to TAP-S medium in the light (A) or in the dark (B) for 0.5h, 1h, 2h, 4h or 6h. Levels of gene transcripts are given as times of relative abundance with respect to the housekeeping control gene (*RACK1*) that has a value of 1. Data are the means±SE from three biological and two technical replicates obtained by real-time RT-PCR. See Supporting experimental procedures (Figures D, E and F and Tables C and D in S1 File) for more information.

Next, we investigated the gene transcription at short time points (Fig 2A). We found that *THB1* mRNA transcript abundance increased about 400-fold after 30 min and reached a maximum of 700-fold induction after 2 h exposure to S deprivation. Furthermore, changes in the *THB1* transcript levels observed in *C*. *reinhardtii* incubated in S-free medium in the dark were very similar to those observed in the light (Fig 2B).

### Isolation of *THB1-ami*RNA strains

**Fig 3:**
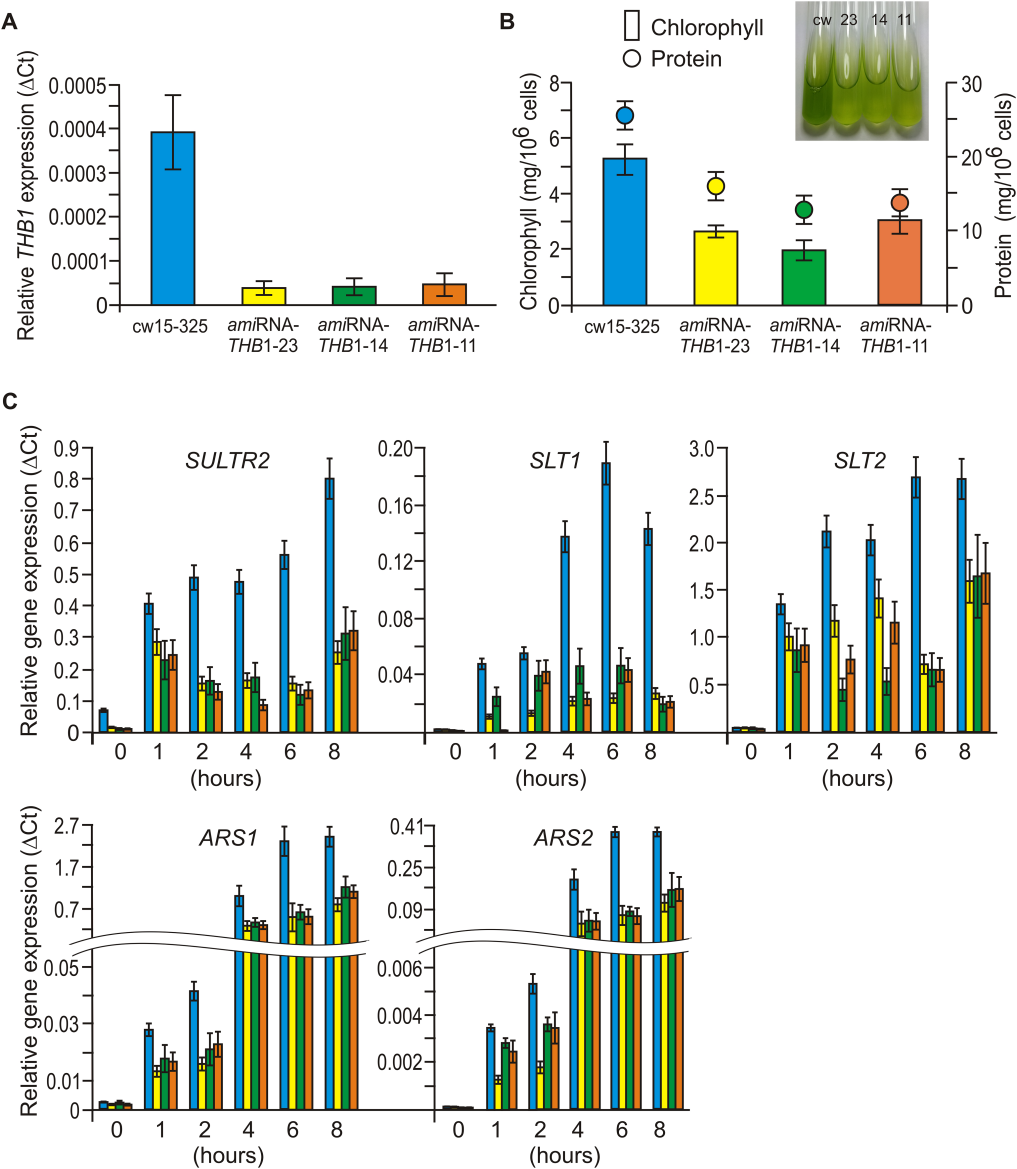
Characterization of *Chlamydomonas reinhardtii THB1* knock-down strains. (A) Real time reverse transcription PCR analysis of *THB1* transcript levels, comparing parental strain cw15-325 to *amiRNA-THB1* strains. Relative expression levels were normalized with the gene expression of *RACK1* and calculated using ΔC_T_; all measurements were done in triplicate. Additional underlying data can be found in S1 Fig. (B) Comparative chlorophyll contents and protein contents of parental strain cw15-325 and *ami*RNA-*THB1* strains. Vegetative cells were grown in TAP medium. Additional underlying data can be found in S2 Fig. Insert shows test tubes with the same cell density of cultures (2 10^6^ cells/ml) in TAP. (C) Expression of selected S limitation-responsive genes in cw15-325 and *ami*RNA-*THB1* strains subjected to S-depleted conditions. Relative expression levels were normalized with the gene expression of *RACK1* and calculated using ΔCt; all measurements were done in triplicate. Cells were treated as described in legend to Fig 2A. See Supporting experimental procedures (Figures G and H and Tables E and F in S1 File) for more information.

For the analysis of the function of the THB1 in *C*. *reinhardtii*, *THB1* underexpression strains demonstrating reduced THB1 transcript amounts were generated with use of the artificial microRNA (amiRNA) approach [35]. The three strains *ami*RNA-*THB1*-23 (10% THB1 expression), *ami*RNA-*THB1*-14 (11.5%) and *ami*RNA-*THB1*-11 (13%) exhibited a significant knock-down of *THB1* mRNA (Fig 3A). After 6 h of incubation in S-free medium, the *ami*RNA-*THB1*-strains had only 12%–25% of *THB1* transcript abundance determined in the parental strain cw15-325 (S1 Fig).

Notably, the knock-down strains had average diameter of 7.1 ± 0.2 μm (namely, 7.3 ± 0.4 μm for *ami*RNA-*THB1*-23, 6.9 ± 0.3 μm for *ami*RNA-*THB1*-14 and 7.1 ± 0.4 μm for *ami*RNA-*THB1*-11), which was smaller than that of the parental strain (8.4 ± 0.54 μm). Moreover, down-regulation of THB1 did affect levels of proteins and chlorophylls (Fig 3B and S2A and S2B Fig). However, the results demonstrated that underexpression of *THB1* was not critical for surviving sulfur deprivation (S2C Fig).

### Consequences of THB1 depletion on the expression of a subset of S limitation-responsive genes

An increased sulfate uptake capacity and the synthesis of extracellular arylsulfatases (ARS) accompany the acclimation of *C*. *reinhardtii* to S limitation [28, 39, 40, 41]. We therefore tested if *THB1* underexpression affected the expression of S deprivation-responsive genes including those encoding the high-affinity sulfate transporters (*SULTR2*, *SLT1* and *SLT2*) and extracellular arylsulfatases (*ARS1* and *ARS2*). As shown in Fig 3C, the downregulation of THB1 impaired the transcription of genes encoding the sulfate transporters in S-free medium: compared with TAP, S deprivation–induced transcript accumulation for *SULTR2*, *SLT1*, *SLT2* genes in all *THB1*-*ami*RNA strains was reduced on average from 4.5-fold to 2.9-fold, 3.7-fold to 8.3-fold and 1.9-fold to 3.0-fold, respectively. Additionally, *THB1*-*ami*RNA cells failed to induce normally *ARS1* and *ARS2* in the absence of S. Our results suggest that THB1 is involved in the control of several genes activation during *C*. *reinhardtii* acclimation to S deprivation.

### S limitation-responsive genes are controlled by NO

**Fig 4:**
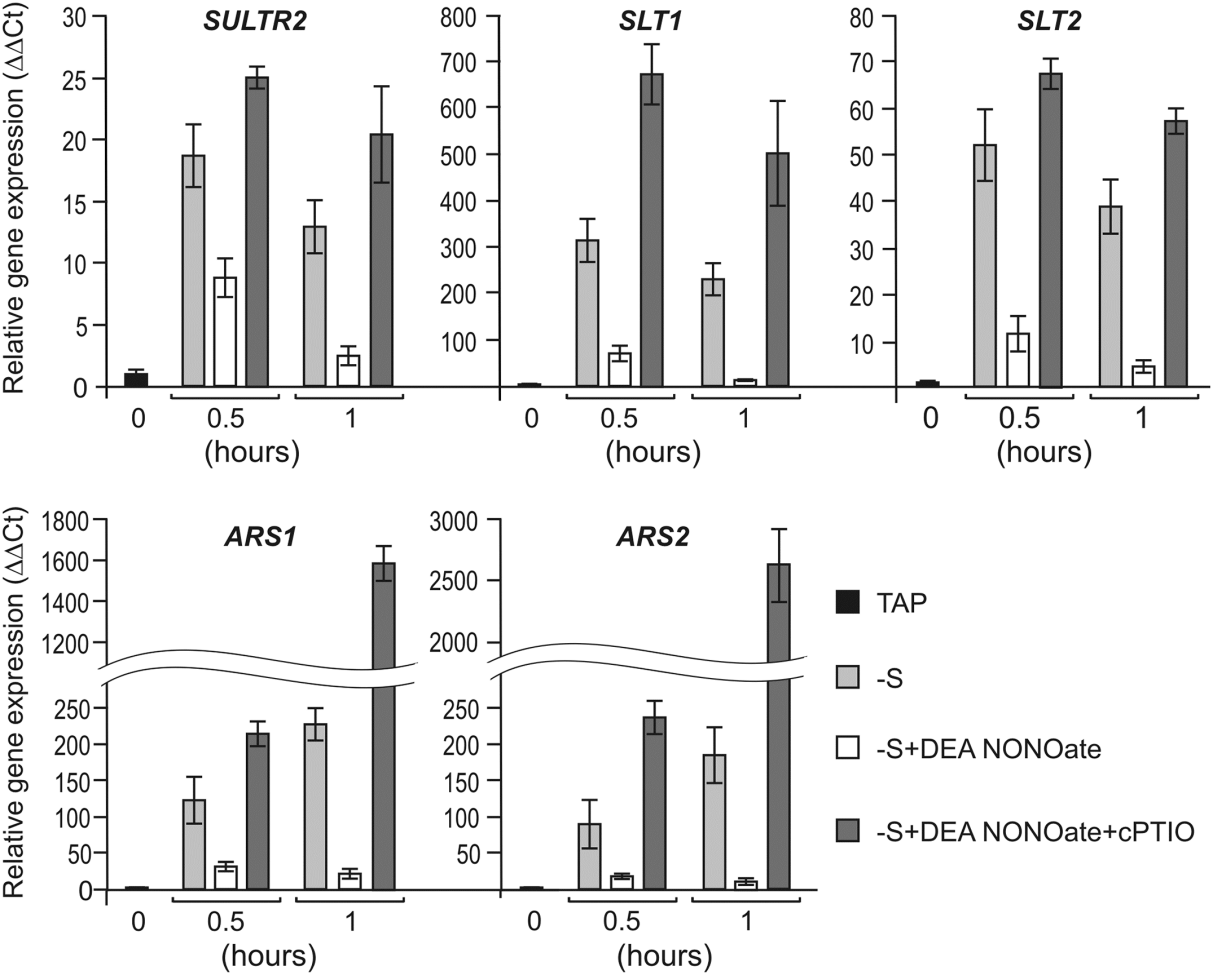
Nitric oxide–dependent expression of selected S limitation-responsive genes in *Chlamydomonas reinhardtii*. Cells grown in TAP were washed in S-free-medium and incubated for 30 min or 1 h in the absence or presence of 50 μM DEA-NONOate. The effect of 100 μM cPTIO was analyzed when added simultaneously with DEA NONOate at time 0. The value 1 was assigned to the expression level of internal standard *RACK1* gene in each condition. Data are the means±SE from three biological and two technical replicates obtained by real-time RT-PCR. See Supporting experimental procedures (Figures I, J and K and Tables G and H in S1 File) for more information.

Recently, it was shown that the THB1 plays a significant role as an NO detoxifier in vivo [18]. To test whether the observed down-regulation of S limitation-responsive genes in *THB1*-*ami*RNA strains resulted from NO overaccumulation, the cells were treated with DEA-NONOate as NO donor [42]. Within 30 min and 1 h following the addition of DEA-NONOate, transcripts of the *SULTR2*, *SLT1*, *SLT2*, *ARS1* and *ARS2* genes were strongly reduced despite the absence of S (Fig 4). As expected, in the presence of the specific NO scavenger cPTIO [43, 44], the expression of these genes was largely recovered. The combined real-time PCR analysis suggested that mRNA levels of these genes were highly controlled by NO.

### Reduction of THB1 enhances NO production under S deprivation

**Fig 5:**
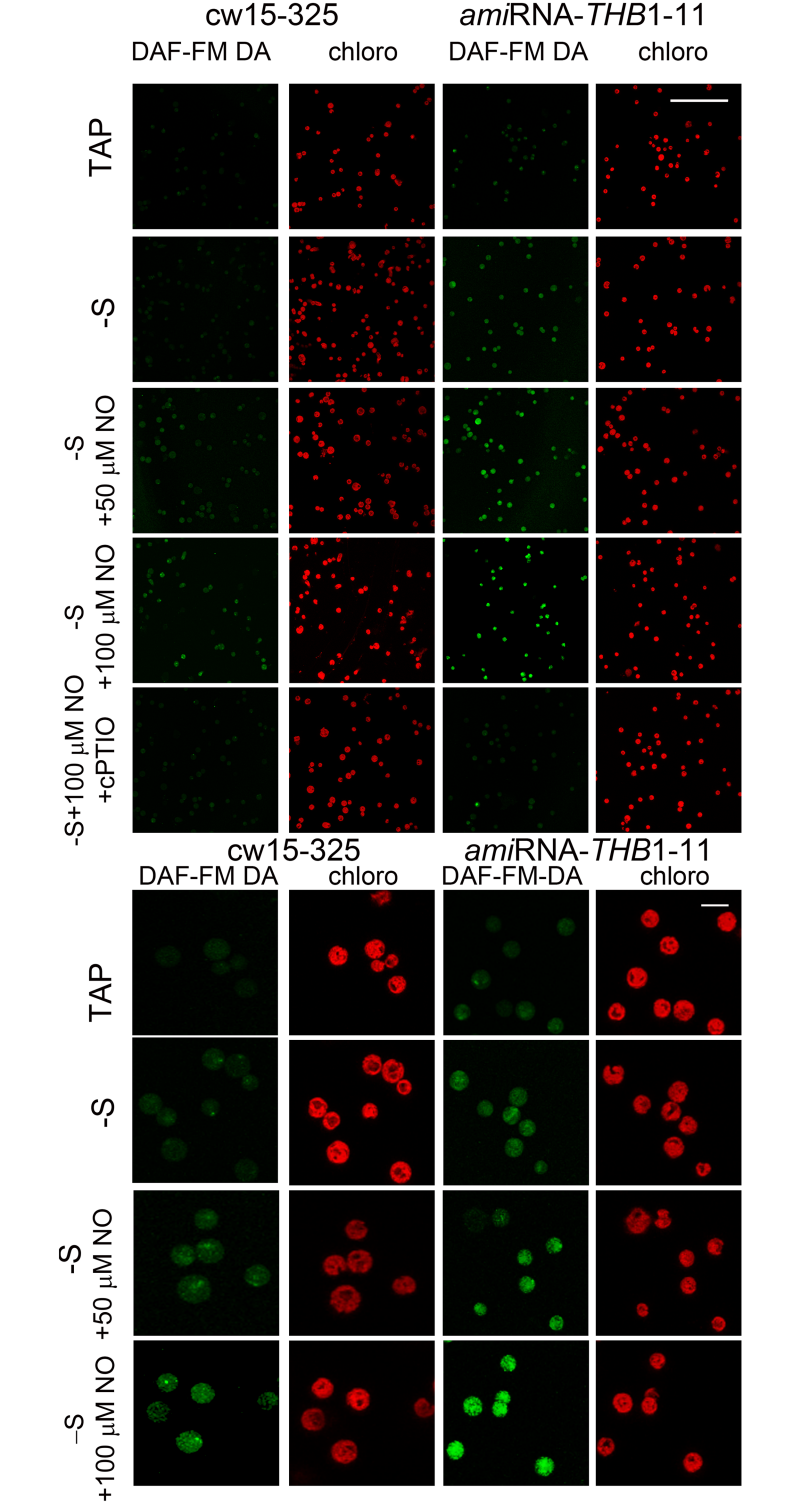
NO visualization in *Chlamydomonas reinhardtii* by confocal microscopy. (A) Images of cell populations grown in TAP (TAP) or incubated in S-free medium (-S) for 15 min. S-deprived cells were treated with 50 μM (-S+50 μM NO) or with 100 μM (-S+100 μM NO) DEA NONOate. Imaging was also performed on the same starved culture supplemented with 100 μM cPTIO and 100 μM DEA NONOate (-S+100 μM NO+cPTIO). The left-hand panels show DAF-FM fluorescence (green color) while the right-hand panels show Chl autofluorescence (red color). Green and red fluorescence images were processed as indicated in Materials and Methods. Scale bar equals 100 μm. Additional underlying data can be found in S3 Fig. (B) Representative cell images. Cw15-325 and *ami*RNA-*THB1*-11 cells were treated as described in legend to (A). Scale bar equals 10 μm.

We next detected intracellular NO production by confocal microscopy with the NO-specific dye DAF-FM DA (Fig 5 and S3 Fig). In TAP medium, cw15-25 and *ami*RNA-*THB1*-11 strains showed either no signal or a very weak one in the cytosol. When these strains were incubated for 15 min in the S-free medium, the percentage of NO-positive cells reached its maximum value of about 15% and 65% for cw15-25 and *ami*RNA-*THB1*-11, respectively (calculated from the data in S3 Fig). In addition, when both strains subjected to S depletion in the presence of 50 μM or 100 μM DEA-NONOate almost 70–85% of green cells were detected. However, green NO fluorescence was different in cw15-25 and *ami*RNA-*THB1*-11 cells kept in S-free TAP in the presence of DEA-NONOate (Fig 5, S3 Fig). Importantly, the fluorescence signal was largely diminished by cPTIO (100 μM).

**Fig 6:**
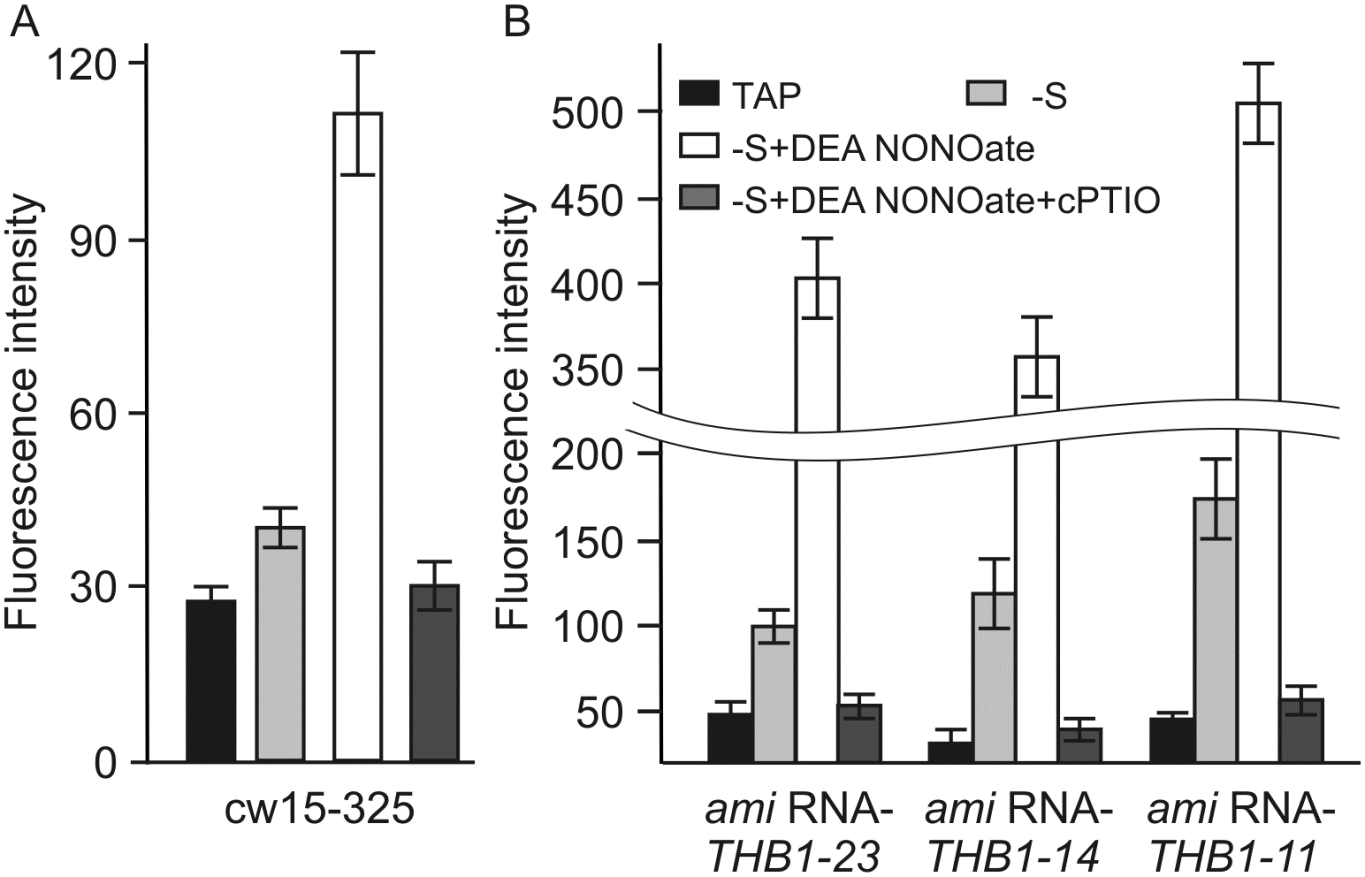
NO production in *Chlamydomonas reinhardtii* following the removal of S from the medium. (A) Vegetative cells of cw15-325 strain were grown in TAP medium and transferred to TAP-S medium in the light for 15 min. Cells were treated with 100 μM DEA NONOate. The effect of 300 μM cPTIO was analyzed when added simultaneously with DEA NONOate at time 0. Fluorescence intensity due to intracellular NO was determined using DAF-FM DA and was expressed as arbitrary units per chlorophyll cells 10^−6^. Cell autofluorescence was subtracted from the total fluorescence obtained. Data are the means±SE from three technical replicates of a representative experiment. (B) Fluorescence increase was measured and expressed in *amiRNA-THB1* cells following the removal of S from the medium as described in (A).

To get a statistical view of NO production, we next quantified intracellular NO formation by spectrofluorometric assays (Fig 6). Cw15-325 cells incubated in TAP medium, showed little accumulation of NO (Fig 6A). In S-free medium, NO formation was slightly higher than in the control medium. Finally, NO donor caused additional increase of a fluorescence signal in cultures deprived of S. The presence of NO signal was largely decreased when cPTIO was added to the medium.

When TAP-grown *THB1*-*ami*RNA cells were incubated for 15 min in S-free medium, a strong fluorescence signal was readily detected (Fig 6B). Most importantly, this level of fluorescence was very similar to the level found in the initial strain in the presence of 100 μM DEA-NANOate (Fig 6A and 6B). Moreover, fluorescence signal appeared to be strongest in the THB1 depleted strains starved for S with 100 μM DEA-NONOate. In addition, DEA-NONOate had no effect after preincubation with cPTIO. The intensities of the overall fluorescence because of NO in the analyzed strains resemble the data in Fig 5. Together, the results demonstrated that the observed misregulation of S deprivation-responsive genes in *THB1*-*ami*RNA strains were probably due to the increase of NO levels above the threshold in the parental strain.

### Sulfur deficiency–induced THB1 optimizes nitrate assimilation

**Fig 7:**
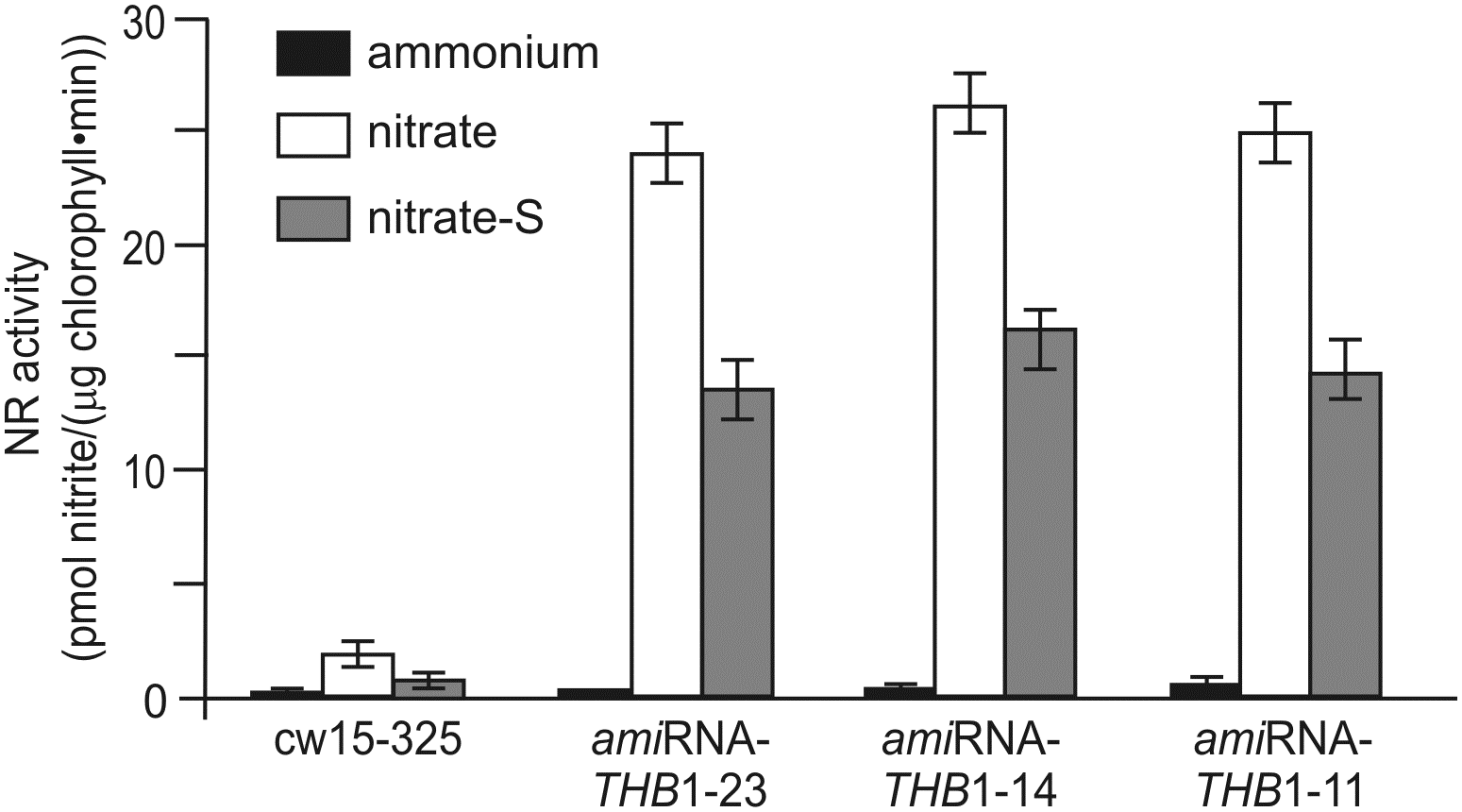
Effects of sulfur depletion on nitrate-induced activity of NR in wild-type and *THB1* knock-down strains. NR was quantified in the cells incubated in TAP containing 8 mM of NH_4_^+^ (ammonium), 4 mM of NO_3_^-^ with S (nitrate) or without S (nitrate-S).

It has been reported previously that THB1 inhibits NR activity [18]. We hypothesize that THB1 not only contributes to acclimation of cells to sulfur deprivation, but also to the nitrate reduction pathway under sulfur starvation, and that through this function it may optimize nitrate assimilation in S-free medium. To examine this hypothesis, wild-type and *ami*RNA-*THB1* transformants, employed for the analysis of NR activity. In 4mM nitrate-exposed wild type cells, NR activity was increased about 18-fold that of the control (Fig 7). THB1 underexpression led to higher NR activity than in parental strain (89-, 92- and 120-fold increase in *ami*RNA-*THB1*-14, *ami*RNA-*THB1*-11 and *ami*RNA-*THB1*-23, respectively). This finding is well consistent with the analysis performed by Sanz-Luque *et al*. [18]. In S-free medium, nitrate-induced activity of NR declined to 36% and 57–62% in cw15-325 and *ami*RNA-*THB1* strains, respectively. It is noteworthy that the enzyme activity was mostly reduced in the parental strain, which had highest levels of *THB1* mRNA (Fig 3A and S1 Fig). Altogether, these data indicate that THB1 is involved in the negative regulation of nitrate assimilation in S-deprived cells.

## Discussion

Truncated hemoglobins are characterized by versatile biological functions in different organisms that are distinct from oxygen delivery and storage [45]. However, a majority of physiological functions of these proteins in plants remains to be elucidated. In this work, we demonstrate that THB1 is linked to the responses to S deprivation.

The TrHbs family in *C*. *reinhardtii* corresponds to the largest one described so far in any organism. One possible reason that can explain the high number of THBs present in *C*. *reinhardtii* could be related to the different functions of these proteins in adaptive responses of unicellular organism to variations in the surrounding conditions. TrHbs in higher plants are induced under hypoxic conditions and might be involved in the adaptation to hypoxia [10, 46]. In *C*. *reinhardtii* one of THBs, THB8, is also essential for anaerobic acclimation of the cells [14]. A number of evidences reveal that another two THBs, THB1 and THB2, are regulated by the nitrogen source [16, 18]. *C*. *reinhardtii* lives both in freshwater and soil, and may encounter various environmental stresses, including nutrients limitation. Because the abundance of S often limits growth, we raised the question whether S-starvation affected the pattern of *THB*s genes expression. Interestingly, three of the twelve *THB* genes (*THB1*, *THB2* and *THB12*) are induced in S-free medium, especially *THB1*, which has the highest expression level (Fig 1). We observed a 700-fold increase upon 2 h of S deprivation (Fig 2A). There was a similar increase upon light-to-dark transition in S-free medium (Fig 2B). Our results strongly suggest that *THB1* is controlled by S-limitation.

To investigate the functional consequences of *THB1* silencing, we generated and characterized three THB1-knock-down strains (Fig 3A and S1 Fig). The *ami*RNA-*THB1* strains displayed obvious phenotype when grown on S-replete or S-deplete media (e.g. cell size, protein and chlorophyll contents were slightly reduced when compared to that of the parental strain) (S2A and S2B Fig). However, the *ami*RNA-*THB1* transformants showed the same viability relative to wild-type cells during S deprivation (S2C Fig). The observed decrease in size, as well as in levels of proteins and chlorophylls suggest for a general reduction of metabolic activity that might be essential for properly cell response to S limitation.

We therefore monitored the kinetics of transcript accumulation of five different genes in three *ami*RNA-*THB1*-strains and cw15-325 during S deprivation (Fig 3C). The genes selected encode extracellular arylsulfatases and high-affinity sulfate transporters that are strongly upregulated under S-limiting conditions [40, 41, 47, 48]. All five genes were inducible by S-deprivation (Fig 3C). However, the abundances of selected transcripts were only about 11–47% compared with wild-type amounts. We conclude that THB1 could be implicated in S limitation-responsive genes expression in these specific conditions.

Why does *C*. *reinhardtii* THB1 impact cell responses to S deprivation? The simplest idea is that THB1 might act by removing the excess of very reactive NO under S limitation. In *C*. *reinhardtii*, THB1 modulates nitric oxide levels [18]. Posttranscriptional silencing of THB1 reduced transcription levels of genes encoding extracellular arylsulfatases and high-affinity sulfate transporters (Fig 3C). Thus, we have studied if NO modulated S-responsive gene expression. The selected genes were strongly repressed in the presence of NO donor (Fig 4). In addition, DEA-NONOate had no reducing effect after incubation with cPTIO. In agreement with these data, confocal microscopy (Fig 5 and S3 Fig) and spectrofluorometric assays (Fig 6) with DAF-FM DA allowed us to detect NO formation in *C*. *reinhardtii* cells starved for S. Furthermore, addition of NO donor in the starvation medium increased the NO amount, whereas NO scavenger had the opposite effect. As expected, *THB1*-*ami*RNA strains showed higher fluorescence levels than parental strain (Figs 5 and 6).

In higher plants, NO is a signaling molecule involved in many physiological processes during plant development and nutrient assimilation. Several recent reports highlighted a role for NO in various signaling pathways in *C*. *reinhardtii*, including cell death [49], remodeling of chloroplast bioenergetics upon nitrogen starvation [50], regulation of nitrate assimilation [18, 51] and anaerobic acclimation [14]. The results obtained suggest that NO also acts as a signaling molecule for the transcriptional regulation of several S-responsive genes upon sulfur deprivation and THB1 is involved in this NO-dependent pathway. In *C*. *reinhardtii*, no animal nitric oxide synthase (NOS) enzyme homolog has been characterized yet. However, NOS-like activity, which is affected by arginine based inhibitors, has been detected enzymatically in the algal cells [51]. This suggests that the structure of the *C*. *reinhardtii* NOS could be unrelated to the animal enzymes. How NO is generated in S-deprived cells when ammonium supplied remains to be elucidated.

Our data begin to clarify a physiological role for *C*. *reinhardtii* THB1 under nutrient limitation conditions. *C*. *reinhardtii* THB1 inhibits NR by uncoupling the electron transfer from NAD(P)H to nitrate [18]. We propose that THB1 might be involved in cross-talk between sulfur deficiency responses and nitrogen metabolism. In higher plants, S limitation resulted in a reduction of NR activity [52, 53]. We have also shown that S depletion led to lower NR activity in parental cells incubated in nitrate-inducing medium (Fig 7). The described regulation of nitrate assimilation by S-limitation is an important mechanism for coordinating the reduction of nitrate with the demand for sulfur. Furthermore, nitrate-induced NR activity correlates negatively with THB1 levels in S-deplete medium (Fig 7). Thus, our data support the idea that in S-deprived cells THB1 plays a dual role in NO detoxification and in coordinating sulfate limitation with nitrate assimilation. Future studies are needed to investigate potential mechanisms of NO production, as well as external cues that modulate THBs induction. This work opens the way to a deeper understanding of the complex pathways that may be regulated and coordinated by truncated hemoglobins.

## Supporting information

S1 Fig Effects of sulfur deprivation conditions on *THB1* transcript accumulation in parental strain cw15-325 and *THB1-ami*RNA strains. Vegetative cells were grown in TAP medium and transferred to TAP-S medium in the light for 1h, 2h, 4h, 6h or 8h. The bars are means of the relative fold change (ΔC_T_) of three biological replicates obtained by real-time RT-PCR. Relative expression levels were normalized with the gene expression of *RACK1*.
(TIF)

S2 Fig Comparative chlorophyll contents (A), protein contents (B) and viability (C) of parental strain cw15-325 and *THB1-ami*RNA strains. Vegetative cells were grown in TAP medium and transferred to TAP-S medium in the light for 24h, 48h, 72h or 96h. A viability dye was used to distinguish viable from nonviable cells as explained in the Materials and Methods section. Values are means ± SD (n = 3).
(TIF)

S3 Fig Confocal images of cell populations from the cw15-325 and *ami*RNA-*THB1*-11 strains grown in TAP (TAP), deprived of S (-S) or deprived of S in the presence of 50 μM (-S+50 μM NO) or 100 μM DEA NONOate (-S+100 μM NO). Imaging was also performed on the same starved culture supplemented with 100 μM cPTIO and 100 μM DEA NONOate (-S+100 μM NO+ cPTIO). The left-hand panels show DAF-FM fluorescence (green color) while the right-hand panels show Chl autofluorescence (red color). Green and red fluorescence images were processed as indicated in Materials and Methods. Scale bar equals 100 μm.
(TIF)

S1 File Gene expression analysis. Fig A. RNA integrity. Electrophoresis of representative RNA samples from *C reinhardtii* cells of cw15-325 strain grown in TAP medium and transferred to TAP-S medium in the light for 8h, 24h, 48h or 72h. Fig B. Melt curve peaks of *THB1-12* and *RACK1*-genes obtained from qRT-PCR analysis. Cells were treated as described in legends to Fig. A. Fig C. Amplification chart of *THB1-12* and *RACK1*-genes obtained from qRT-PCR analysis. Cells were treated as described in legends to Fig. A. Profiling experiments were performed in 96-well plates. Table A. The Ct values across replicates in *C reinhardtii* cells of cw15-325 strain grown in TAP medium and transferred to TAP-S medium in the light for 8h, 24h, 48h or 72h. Table B. Relative *THB1-12* gene expression in *C reinhardtii* cells of cw15-325 strain grown in TAP medium and transferred to TAP-S medium in the light for 8h, 24h, 48h or 72h. Fig D. RNA integrity. Electrophoresis of representative RNA samples from light-grown *C*. *reinhardtii* cw15-325 cells that were transferred to TAP-S medium in the light (A) or in the dark (B) for 0.5h, 1h, 2h, 4h or 6h. Fig E. Semi-quantitative RT-PCR analysis with *THB1* and *RACK1* specific primers (A) and melt curve peaks of *THB1* and *RACK1* genes obtained from qRT-PCR (B, C). Cells in the light (B) or in the dark (C) were treated as described in legends to Fig. D. Fig F. Amplification chart of *THB1* and *RACK1* genes obtained from qRT-PCR analysis. Cells were treated as described in legends to Fig. E. Table C. The Ct values across replicates in S-deprived *C*. *reinhardtii* cw15-325 cells incubated in the light or in the dark for 0.5h, 1h, 2h, 4h or 6h. Table D. Comparison of relative THB1 expression in S-deprived *C*. *reinhardtii* cw15-325 cells incubated in the light or in the dark for 0.5h, 1h, 2h, 4h or 6h. Fig G. RNA integrity. Electrophoresis of representative RNA samples from *THB1* knock-down strains grown in TAP medium and transferred to TAP-S medium in the light for 1h, 2h, 4h, 6h or 8h. Fig H. Amplification chart of *ARS1*, *ARS2*, *SLT1*, *SLT2* and *SULTR2* genes obtained from qRT-PCR analysis. Cells were treated as described in legends to Fig. G. Table E. The Ct values for *ARS1*, *ARS2*, *SLT1*, *SLT2* and *SULTR2* genes across replicates in S-deprived *C*. *reinhardtii* cw15-325 and three *ami*THB1 strains incubated in the light for 1h, 2h, 4h, 6h or 8h. Table E. The Ct values for *ARS1*, *ARS2*, *SLT1*, *SLT2* and *SULTR2* genes across replicates in S-deprived *C*. *reinhardtii* cw15-325 and three *ami*THB1 strains incubated in the light for 1h, 2h, 4h, 6h or 8h. Table F. Comparison of relative expression (ΔCt) in S-deprived *C*. *reinhardtii* cw15-325 and three *ami*THB1 strains incubated in the light for 1h, 2h, 4h, 6h or 8h. Fig I. RNA integrity. Electrophoresis of representative RNA samples from *C*. *reinhardtii* cw15-325 cells grown in TAP, washed in S-free-medium (-S) and incubated for 0.5 h or 1 h in the absence or presence of 50 μM DEA-NONOate (NO) with or without 100 μM cPTIO. Fig J. Melt curve peaks of *ARS1*, *ARS2*, *SLT1*, *SLT2* and *SULTR2* genes obtained from qRT-PCR analysis. Cells were treated as described in legends to Fig. I. Fig. K. Amplification chart of *ARS1*, *ARS2*, *SLT1*, *SLT2* and *SULTR2* genes obtained from qRT-PCR analysis. Cells were treated as described in legends to Fig. I. Table G. The Ct values for *ARS1*, *ARS2*, *SLT1*, *SLT2* and *SULTR2* genes across replicates in S-deprived *C*. *reinhardtii* cw15-325 cells incubated for 0.5 h or 1 h in the absence or presence of 50 μM DEA-NONOate (NO) with or without 100 μM cPTIO. Table H. Comparison of relative *ARS1*, *ARS2*, *SLT1*, *SLT2* and *SULTR2* expression in S-deprived *C*. *reinhardtii* cw15-325 cells incubated for 0.5 h or 1 h in the absence or presence of 50 μM DEA-NONOate (NO) with or without 100 μM cPTIO.
(PDF)

S1 Table Primer list. (DOCX)
