# Supplementary figures and images for: Truncated hemoglobin 1 is a new player in Chlamydomonas reinhardtii acclimation to sulfur deprivation
Source: PLoS One. 2017 Oct 19;12(10):e0186851. doi: 10.1371/journal.pone.0186851 (PMC5648252; doi:10.1371/journal.pone.0186851)

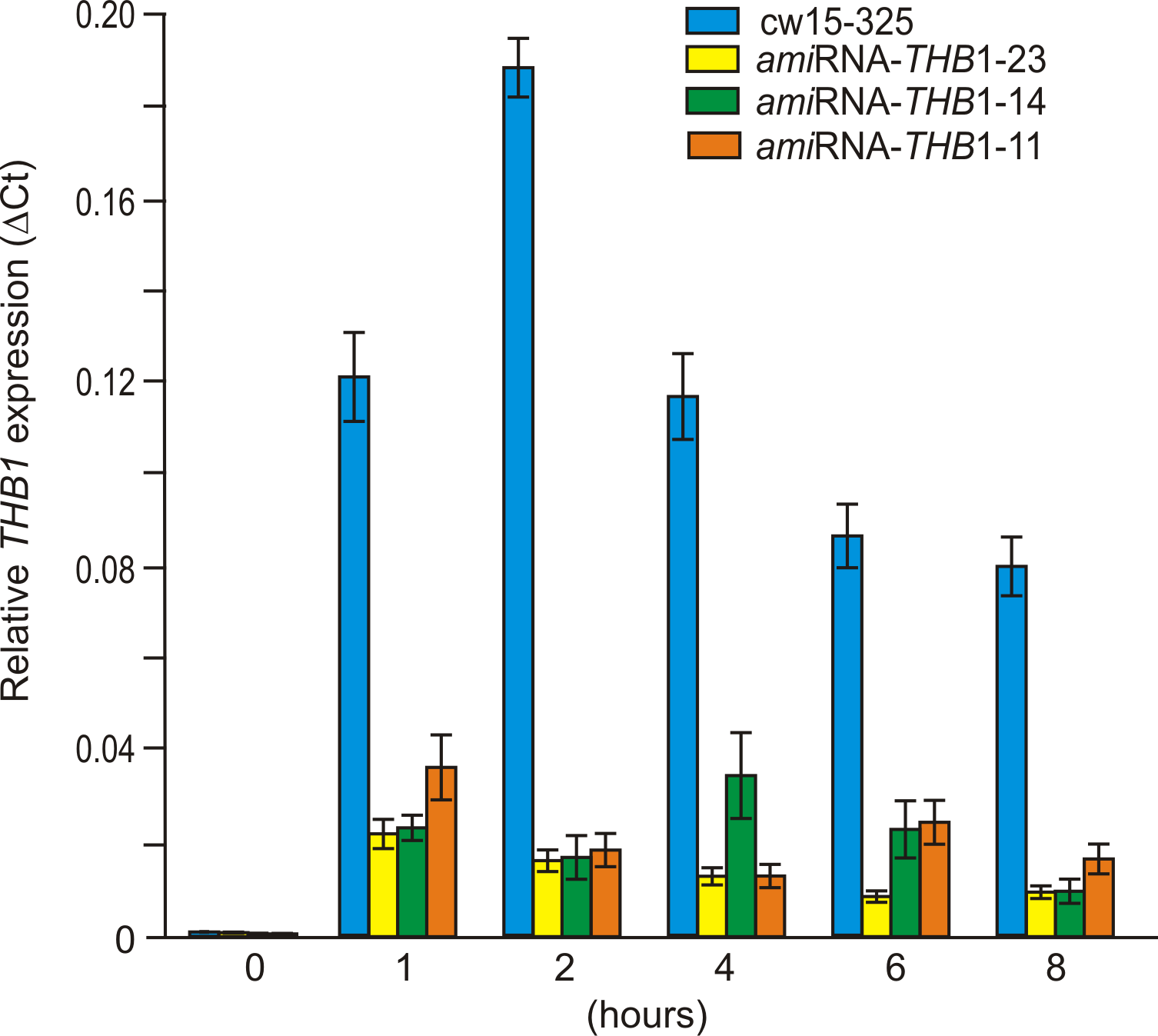

Supplement: S1 Fig — Vegetative cells were grown in TAP medium and transferred to TAP-S medium in the light for 1h, 2h, 4h, 6h or 8h. The bars are means of the relative fold change (ΔCT) of three biological replicates obtained by real-time RT-PCR. Relative expression levels were normalized with the gene expression of RACK1. (TIF) [file pone.0186851.s001.tif]

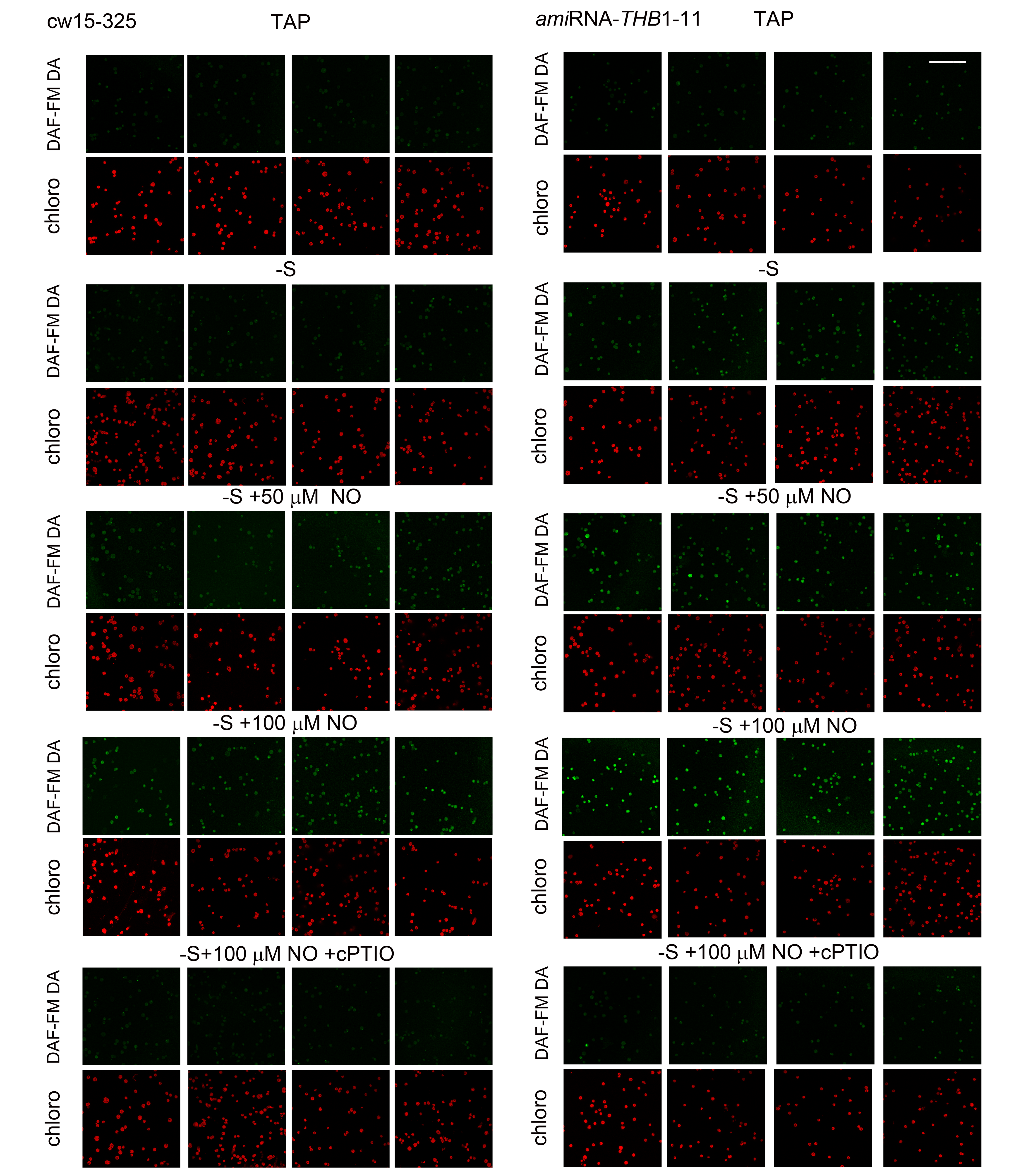

Supplement: S3 Fig — Imaging was also performed on the same starved culture supplemented with 100 μM cPTIO and 100 μM DEA NONOate (-S+100 μM NO+ cPTIO). The left-hand panels show DAF-FM fluorescence (green color) while the right-hand panels show Chl autofluorescence (red color). Green and red fluorescence images were processed as indicated in Materials and Methods. Scale bar equals 100 μm. (TIF) [file pone.0186851.s003.tif]
