# Supplementary material for: Truncated hemoglobin 1 is a new player in Chlamydomonas reinhardtii acclimation to sulfur deprivation
Source: PLoS One. 2017 Oct 19;12(10):e0186851. doi: 10.1371/journal.pone.0186851 (PMC5648252; doi:10.1371/journal.pone.0186851)
Supplement: S1 File — Fig A. RNA integrity. Electrophoresis of representative RNA samples from C reinhardtii cells of cw15-325 strain grown in TAP medium and transferred to TAP-S medium in the light for 8h, 24h, 48h or 72h. Fig B. Melt curve peaks of THB1-12 and RACK1-genes obtained from qRT-PCR analysis. Cells were treated as described in legends to Fig. A. Fig C. Amplification chart of THB1-12 and RACK1-genes obtained from qRT-PCR analysis. Cells were treated as described in legends to Fig. A. Profiling experiments were performed in 96-well plates. Table A. The Ct values across replicates in C reinhardtii cells of cw15-325 strain grown in TAP medium and transferred to TAP-S medium in the light for 8h, 24h, 48h or 72h. Table B. Relative THB1-12 gene expression in C reinhardtii cells of cw15-325 strain grown in TAP medium and transferred to TAP-S medium in the light for 8h, 24h, 48h or 72h. Fig D. RNA integrity. Electrophoresis of representative RNA samples from light-grown C. reinhardtii cw15-325 cells that were transferred to TAP-S medium in the light (A) or in the dark (B) for 0.5h, 1h, 2h, 4h or 6h. Fig E. Semi-quantitative RT-PCR analysis with THB1 and RACK1 specific primers (A) and melt curve peaks of THB1 and RACK1 genes obtained from qRT-PCR (B, C). Cells in the light (B) or in the dark (C) were treated as described in legends to Fig. D. Fig F. Amplification chart of THB1 and RACK1 genes obtained from qRT-PCR analysis. Cells were treated as described in legends to Fig. E. Table C. The Ct values across replicates in S-deprived C. reinhardtii cw15-325 cells incubated in the light or in the dark for 0.5h, 1h, 2h, 4h or 6h. Table D. Comparison of relative THB1 expression in S-deprived C. reinhardtii cw15-325 cells incubated in the light or in the dark for 0.5h, 1h, 2h, 4h or 6h. Fig G. RNA integrity. Electrophoresis of representative RNA samples from THB1 knock-down strains grown in TAP medium and transferred to TAP-S medium in the light for 1h, 2h, 4h, 6h or 8h. Fig H. Amplification [file pone.0186851.s004.pdf]

S1 File. Gene expression analysis

Fig A. RNA integrity

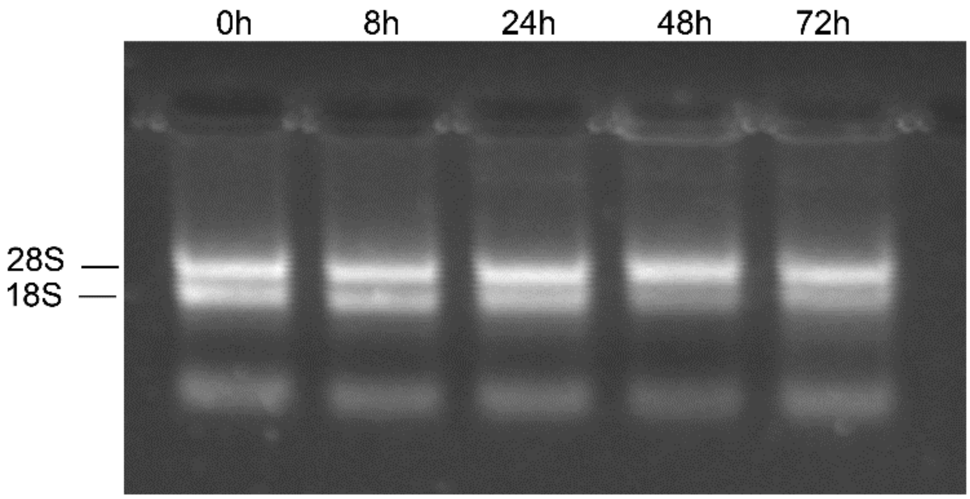

.

Fig B. Melt curve peaks of *THB1-12* and *RACK1*-genes obtained from qRT-PCR analysis.

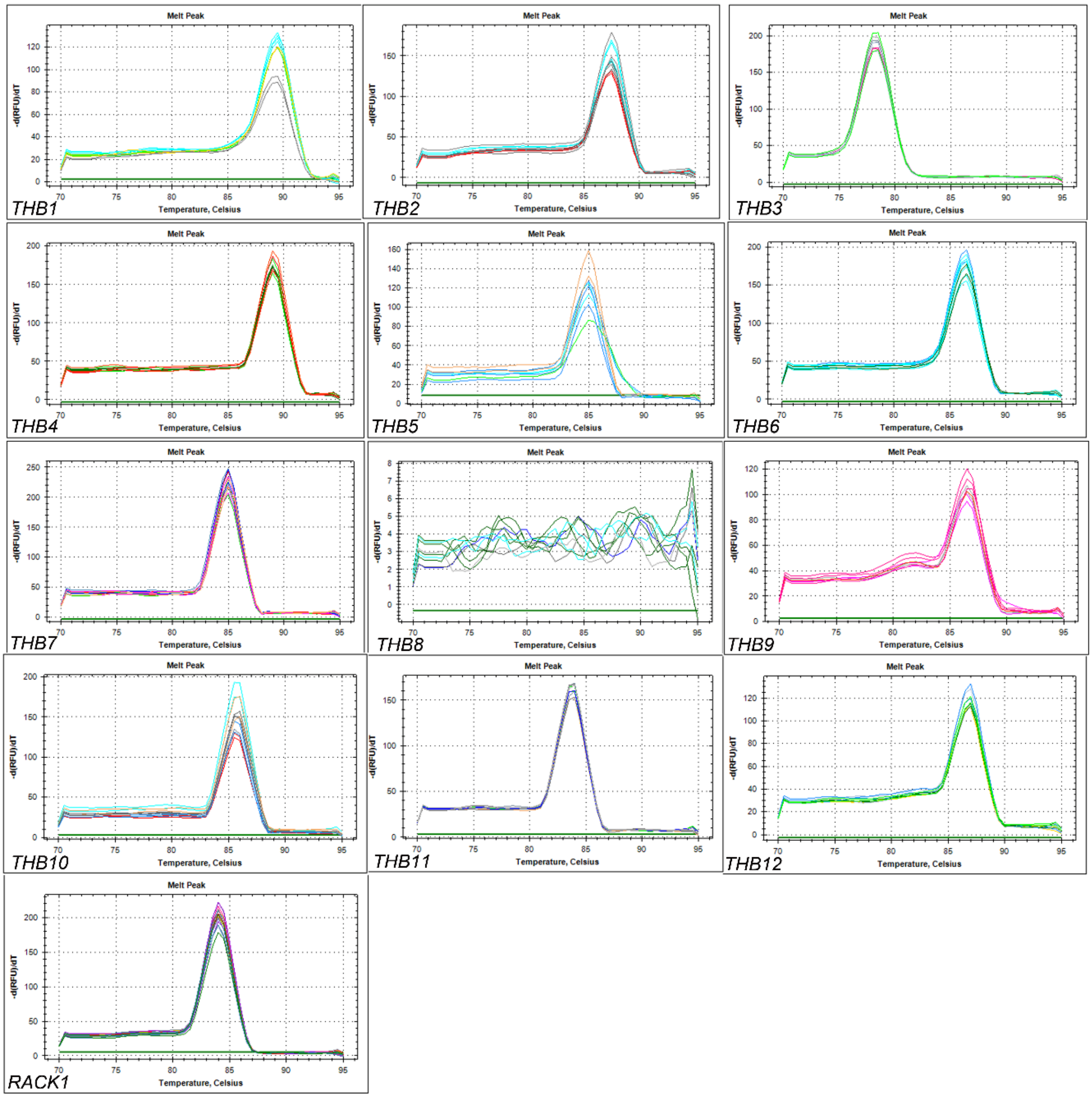

Fig C. Amplification chart of *THB1-12* and *RACK1*-genes obtained from qRT-PCR analysis.

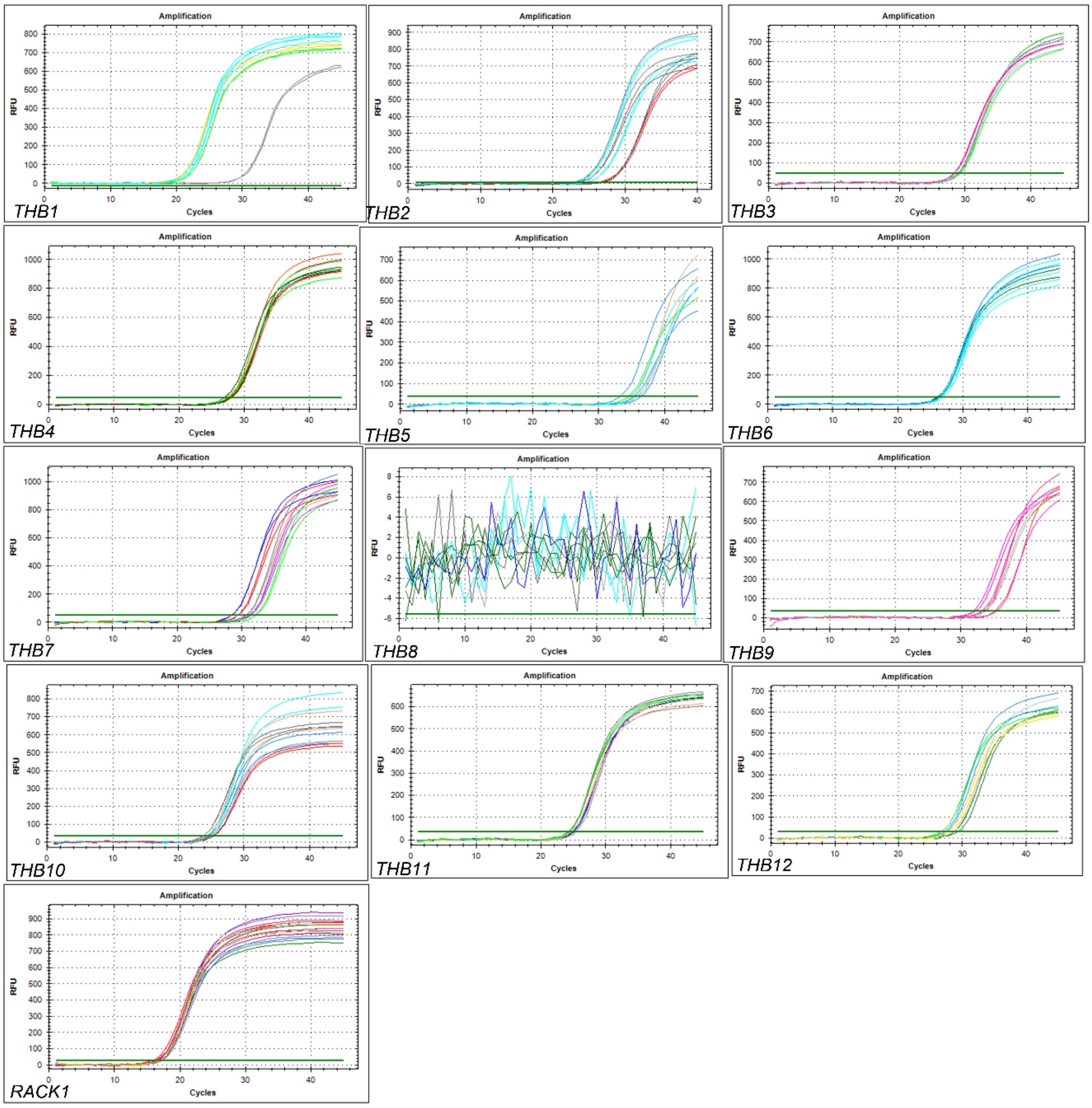

Table A. The Ct values across replicates in *C reinhardtii* cells of cw15-325 strain grown in TAP medium and transferred to TAP-S medium in the light for 8h, 24h, 48h or 72h.

Variant	Replicate	Gene	Theshold cycle (C_T)	C_T mean
TAP	1	*THB1*	31.42	31.33
	1		31.24	
	1		31.33	
	1	*THB2*	30.03	29.93
	1		29.80	
	1		29.93	
	1	*THB3*	28.34	28.32
	1		28.21	
	1		28.40	
	1	*THB4*	26.95	27.04
	1		27.01	
	1		27.15	
	1	*THB5*	34.42	34.57
	1		34.74	
	1		34.55	
	1	*RACK1*	18.55	17.50
	1		18.45	
	1		18.51	
	1	*THB6*	26.24	26.25
	1		26.20	
	1		26.31	
	1	*THB7*	29.46	29.41
	1		29.28	
	1		29.50	
	1	*THB9*	34.69	34.62
	1		34.55	
	1		34.62	
	1	*THB10*	26.22	26.00
	1		25.79	
	1		26.00	
	1	*THB11*	26.15	26.03
	1		26.09	
	1		25.86	
		RACK1	17.55	17.45
	1		17.42	
	1		17.38	
	1	*THB12*	37.22	37.22
	1		37.11	
	1		37.34	
	1	*RACK1*	17.45	17.53
	1		17.59	
	1		17.55	
-S 8h	2	*THB1*	22.64	22.51

	2		22.49	
	2		22.40	
	2	*THB2*	26.11	26.01
	2		25.94	
	2		25.99	
	2	*THB3*	29.14	29.20
	2		29.26	
	2		29.20	
	2	*THB4*	26.65	26.72
	2		26.87	
	2		26.63	
	2	*THB5*	34.94	35.12
	2		35.25	
	2		35.17	
	2	*RACK1*	18.34	17.30
	2		18.35	
	2		18.21	
	2	*THB6*	27.16	27.15
	2		27.02	
	2		27.27	
	2	*THB7*	30.14	30.18
	2		30.32	
	2		30.09	
	2	*THB9*	34.98	34.84
	2		34.69	
	2		34.85	
	2	*THB10*	26.74	26.68
	2		26.62	
	2		26.68	
	2	*THB11*	25.19	25.18
	2		25.28	
	2		25.07	
	2	*RACK1*	17.30	17.32
	2		17.41	
	2		17.26	
	2	*THB12*	29.34	29.22
	2		29.12	
	2		29.19	
	2	*RACK1*	17.34	17.35
	2		17.46	
	2		17.24	
-S 24h	3	*THB1*	23.19	23.12
	3		23.01	
	3		23.15	
	3	*THB2*	24.88	24.75
	3		24.65	

	3		24.72	
	3	*THB3*	28.99	28.98
	3		28.86	
	3		29.08	
	3	*THB4*	27.78	27.61
	3		27.45	
	3		27.61	
	3	*THB5*	35.02	34.92
	3		34.83	
	3		34.92	
	3	*RACK1*	18.83	17.83
	3		18.79	
	3		18.87	
	3	*THB6*	26.99	26.91
	3		26.82	
	3		26.91	
	3	*THB7*	31.41	31.30
	3		31.19	
	3		31.30	
	3	*THB9*	35.84	35.87
	3		36.00	
	3		35.78	
	3	*THB10*	26.53	26.41
	3		26.29	
	3		26.41	
	3	*THB11*	25.57	25.72
	3		25.89	
	3		25.71	
	3	*RACK1*	17.79	17.79
	3		17.87	
	3		17.70	
	3	*THB12*	29.92	29.89
	3		29.75	
	3		29.99	
	3	*RACK1*	17.74	17.74
	3		17.81	
	3		17.68	
-S 48h	4	*THB1*	22.96	22.96
	4		23.05	
	4		22.87	
	4	*THB2*	25.89	25.85
	4		25.74	
	4		25.91	
	4	*THB3*	29.11	29.00
	4		29.02	
	4		28.87	

	4	*THB4*	27.66	27.59
	4		27.49	
	4		27.62	
	4	*THB5*	34.04	34.04
	4		33.97	
	4		34.11	
	4	*RACK1*	18.75	17.79
	4		18.74	
	4		18.87	
	4	*THB6*	26.96	26.99
	4		27.12	
	4		26.89	
	4	*THB7*	31.15	30.96
	4		30.82	
	4		30.91	
	4	*THB9*	35.29	35.08
	4		34.87	
	4		35.09	
	4	*THB10*	26.38	26.34
	4		26.43	
	4		26.21	
	4	*THB11*	25.87	25.89
	4		25.81	
	4		25.98	
	4	*RACK1*	17.85	17.83
	4		17.75	
	4		17.89	
	4	*THB12*	30.92	31.02
	4		31.11	
	4		31.04	
	4	*RACK1*	17.61	17.59
	4		17.52	
	4		17.64	
-S 72h	5	*THB1*	22.87	22.79
	5		22.69	
	5		22.80	
	5	*THB2*	27.22	27.17
	5		27.11	
	5		27.17	
	5	*THB3*	29.11	29.06
	5		29.00	
	5		29.06	
	5	*THB4*	27.88	28.03
	5		28.08	
	5		28.12	
	5	*THB5*	33.93	33.82

	5		33.71	
	5		33.82	
	5	*RACK1*	18.88	17.88
	5		18.96	
	5		18.79	
	5	*THB6*	27.33	27.43
	5		27.48	
	5		27.49	
	5	*THB7*	32.19	32.16
	5		32.09	
	5		32.21	
	5	*THB9*	35.09	35.18
	5		35.22	
	5		35.24	
	5	*THB10*	27.02	26.92
	5		26.84	
	5		26.91	
	5	*THB11*	25.99	25.89
	5		25.72	
	5		25.97	
	5	*RACK1*	17.82	17.85
	5		17.95	
	5		17.79	
	5	*THB12*	30.16	30.29
	5		30.43	
	5		30.29	
	5	*RACK1*	17.72	17.79
	5		17.88	
	5		17.78	

Table B. Relative *THB1-12* gene expression in *C reinhardtii* cells of cw15-325 strain grown in TAP medium and transferred to TAP-S medium in the light for 8h, 24h, 48h or 72h.

Variant	Average C_T	Average C_T	ΔC_T	$\Delta\Delta C_T$ (Avg ΔC_T –	$2^{-\Delta\Delta C_T}$

	THB	*RACK1*	(Avg *THB* C_T – Avg *RACK1* C_T)	Avg ΔC_T TAP)	
THB1					
TAP	31.33±0.09	17.50±0.05	13.83		1
–S 8h	22.51±0.12	17.30±0.08	5.21	-8.62	394.48
–S 24h	23.12±0.09	17.83±0.04	5.29	-8.54	373.20
–S 48h	22.96±0.09	17.79±0.07	5.17	-8.66	405.57
–S 72h	22.79±0.09	17.88±0.09	4.91	-8.92	485.67
THB2					
TAP	29.93±0.10	17.50±0.05	12.43		1
–S 8h	26.01±0.09	17.30±0.08	8.71	-3.72	13.18
–S 24h	24.75±0.12	17.83±0.04	6.92	-5.51	45.57
–S 48h	25.85±0.09	17.79±0.07	8.06	-4.37	20.68
–S 72h	27.17±0.06	17.88±0.09	9.29	-3.14	8.82
THB3					
TAP	28.32±0.10	17.50±0.05	10.82		1
–S 8h	29.20±0.06	17.30±0.08	11.90	1.08	0.47
–S 24h	28.98±0.11	17.83±0.04	11.15	0.33	0.80
–S 48h	29.00±0.12	17.79±0.07	11.21	0.39	0.76
–S 72h	29.06±0.06	17.88±0.09	11.18	0.36	0.78
THB4					
TAP	27.04±0.10	17.50±0.05	9.54		1
–S 8h	26.72±0.13	17.30±0.08	9.42	-0.12	1.09
–S 24h	27.61±0.17	17.83±0.04	9.78	0.24	0.85
–S 48h	27.59±0.09	17.79±0.07	9.80	0.26	0.84
–S 72h	28.03±0.13	17.88±0.09	10.15	0.61	0.66
THB5					
TAP	34.57±0.16	17.50±0.05	17.07		1
–S 8h	35.12±0.16	17.30±0.08	17.82	0.75	0.60

–S 24h	34.92±0.10	17.83±0.04	17.09	0.01	0.99
–S 48h	34.04±0.07	17.79±0.07	16.25	-0.83	1.78
–S 72h	33.82±0.11	17.88±0.09	15.94	-1.14	2.20
THB6					
TAP	26.25±0.06	17.45±0.09	8.80		1
–S 8h	27.15±0.13	17.32±0.08	9.83	1.03	0.49
–S 24h	26.91±0.09	17.79±0.09	9.12	0.32	0.80
–S 48h	26.99±0.12	17.83±0.07	9.16	0.36	0.78
–S 72h	27.43±0.09	17.85±0.09	9.58	0.78	0.58
THB7					
TAP	29.41±0.12	17.45±0.09	11.96		1
–S 8h	30.18±0.12	17.32±0.08	12.86	0.90	0.535
–S 24h	31.30±0.11	17.79±0.09	13.51	1.55	0.341
–S 48h	30.96±0.17	17.83±0.07	13.13	1.17	0.445
–S 72h	32.16±0.06	17.85±0.09	14.31	2.35	0.196
THB9					
TAP	34.62±0.07	17.45±0.09	17.17		1
–S 8h	34.84±0.15	17.32±0.08	17.53	0.36	0.78
–S 24h	35.87±0.11	17.79±0.09	18.08	0.91	0.53
–S 48h	35.08±0.21	17.83±0.07	17.23	0.06	0.96
–S 72h	35.18±0.08	17.85±0.09	17.33	0.16	0.89
THB10					
TAP	26.00±0.22	17.45±0.09	8.55		1
–S 8h	26.68±0.06	17.32±0.08	9.36	0.81	0.57
–S 24h	26.41±0.12	17.79±0.09	8.62	0.06	0.96
–S 48h	26.34±0.12	17.83±0.07	8.51	-0.04	1.03
–S 72h	26.92±0.09	17.85±0.09	9.07	0.52	0.70
THB11					

TAP	26.03±0.15	17.45±0.09	8.58		1
–S 8h	25.18±0.11	17.32±0.08	7.86	-0.72	1.64
–S 24h	25.72±0.16	17.79±0.09	7.93	-0.65	1.56
–S 48h	25.89±0.09	17.83±0.07	8.06	-0.52	1.43
–S 72h	25.89±0.15	17.85±0.09	8.04	-0.54	1.45
THB12					
TAP	32.22±0.12	17.53±0.07	14.69		1
–S 8h	29.22±0.11	17.35±0.11	11.85	-2.84	7.14
–S 24h	29.89±0.12	17.74±0.07	12.15	-2.54	5.82
–S 48h	31.02±0.10	17.59±0.06	13.43	-1.26	2.39
–S 72h	30.29±0.14	17.79±0.08	12.50	-2.19	4.57

$\Delta\Delta C_T$= (C_T, gene of interest– C_T, *RACK1*)$_{\text{Time X}}$ – (C_T, gene of interest – C_T, *RACK1*)$_{\text{Time 0}}$.

Fig D. RNA integrity.

A Light

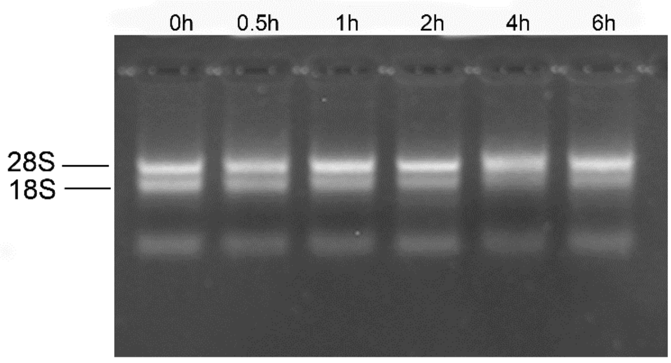

B Dark

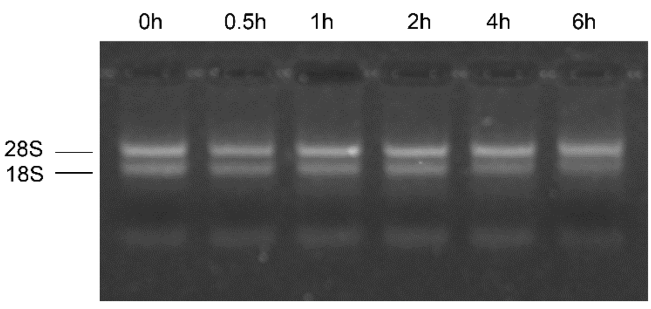

Fig E. Semi-quantitative RT-PCR analysis with *THB1* and *RACK1* specific primers (A) and melt curve peaks of *THB1* and *RACK1* genes obtained from qRT-PCR (B, C).

A

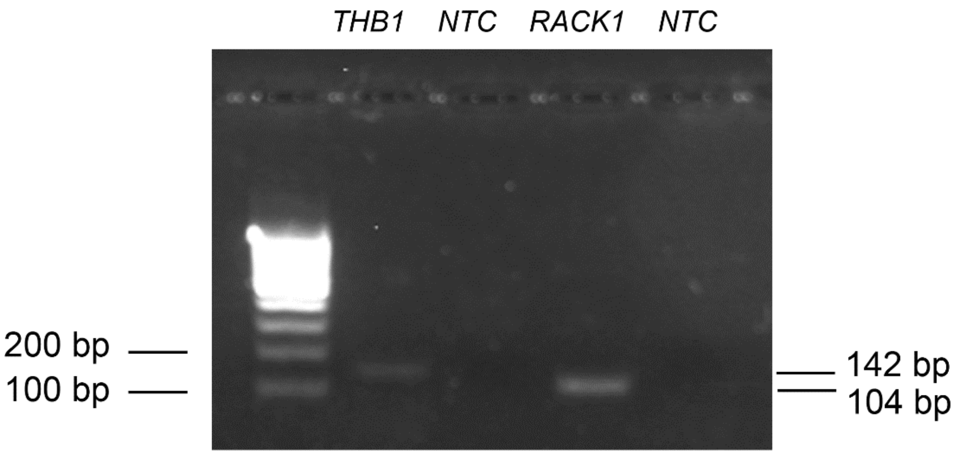

NTC - no template control

100 bp DNA Ladder (Cat. #M15 http://www.sibenzyme.com/info410.php)

B

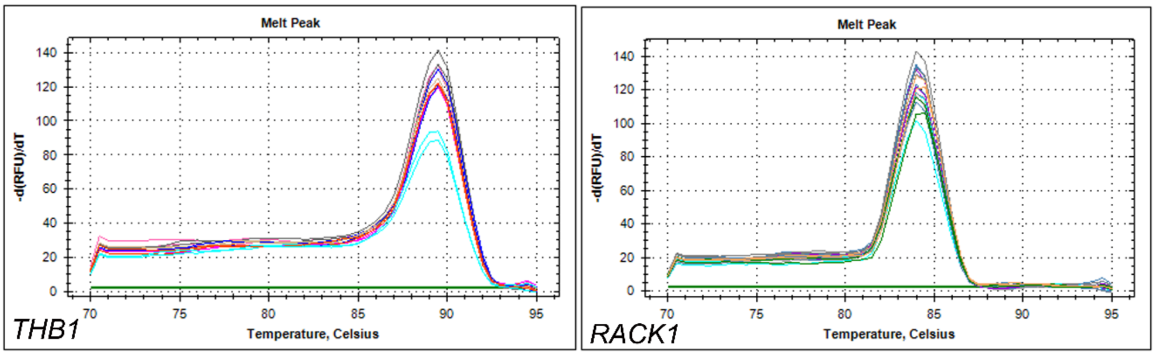

C

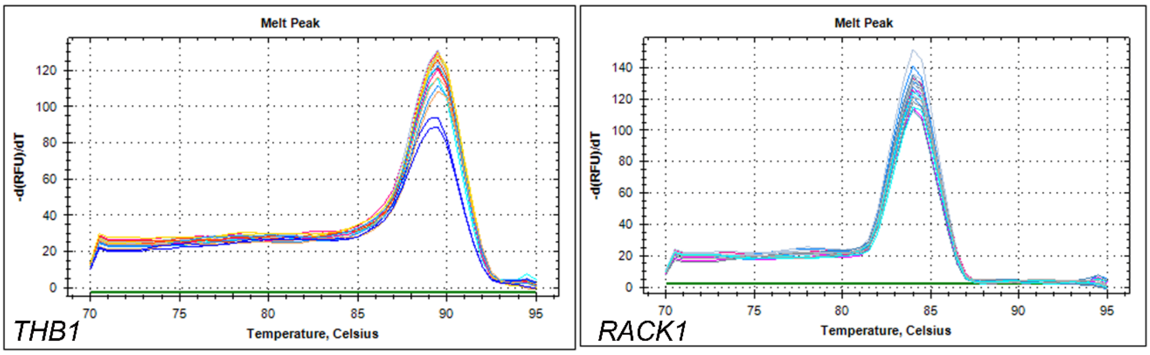

Fig F. Amplification chart of *THB1* and *RACK1* genes obtained from qRT-PCR analysis. Cells were treated as described in legends to Fig. E.

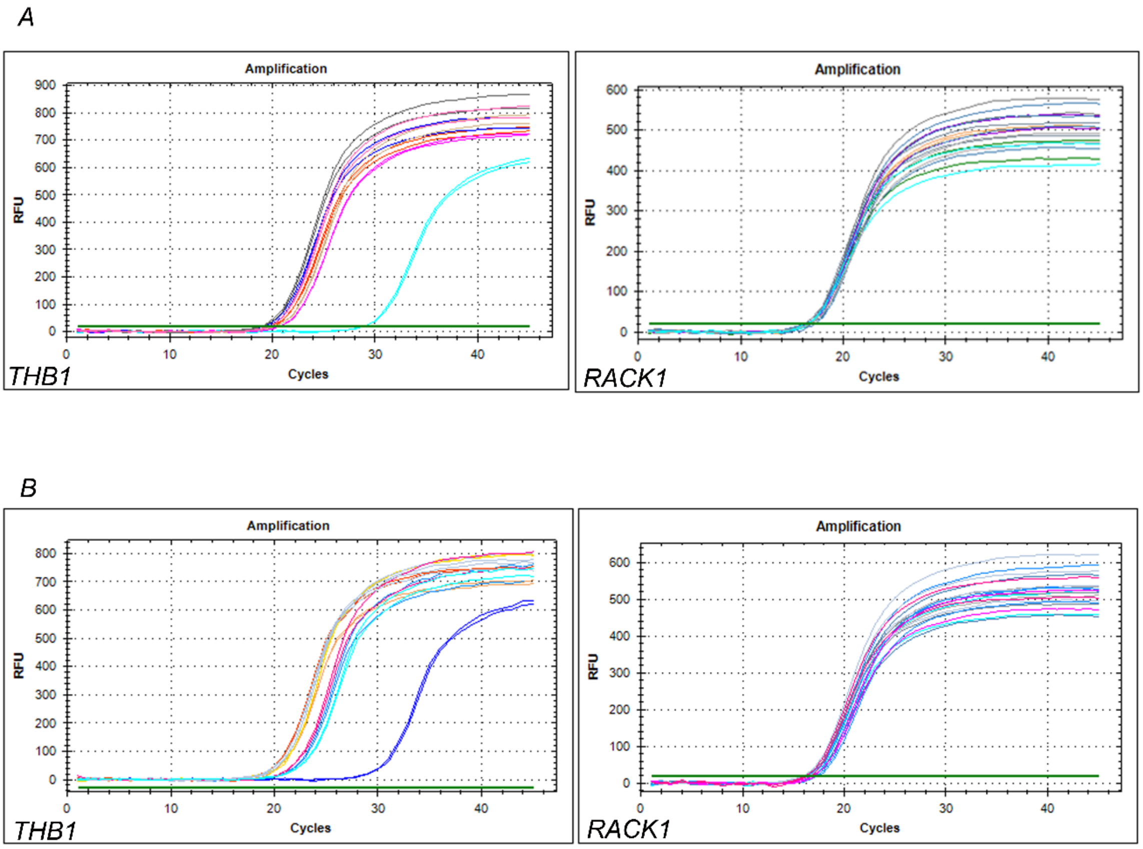

Table C. The Ct values across replicates in S-deprived *C. reinhardtii* cw15-325 cells incubated in the light or in the dark for 0.5h, 1h, 2h, 4h or 6h.

Variant	Replicate	Gene	Theshold cycle (C_T)	C_T mean
TAP	1	*THB1*	31.37	31.25
	1		31.19	
	1		31.18	
	1	*RACK1*	18.44	18.43
	1		18.44	
	1		18.41	
–S 0.5h light	2	*THB1*	22.84	22.76
	2		22.71	
	2		22.72	
	2	*RACK1*	18.82	18.80
	2		18.79	
	2		18.78	
–S 1h light	3	*THB1*	22.37	22.50
	3		22.61	
	3		22.51	
	3	*RACK1*	18.86	18.83
	3		18.81	
	3		18.82	
–S 2h light	4	*THB1*	22.54	22.39
	4		22.31	
	4		22.32	
	4	*RACK1*	19.14	19.12
	4		19.09	
	4		19.14	
–S 4h light	5	*THB1*	22.76	22.73

	5		22.70	
	5		22.72	
	5	*RACK1*	18.86	18.82
	5		18.80	
	5		18.79	
–S 6h light	6	*THB1*	23.04	23.08
	6		23.18	
	6		23.03	
	6	*RACK1*	18.81	18.76
	6		18.75	
	6		18.73	
–S 0.5h dark	7	*THB1*	22.19	22.19
	7		22.03	
	7		22.35	
	7	*RACK1*	18.52	18.51
	7		18.52	
	7		18.50	
–S 1h dark	8	*THB1*	22.16	22.18
	8		22.23	
	8		22.16	
	8	*RACK1*	18.71	18.69
	8		18.70	
	8		18.67	
–S 2h dark	9	*THB1*	22.49	22.51
	9		22.46	
	9		22.59	
	9	*RACK1*	19.21	19.15
	9		19.11	
	9		19.14	

–S 4h dark	10	*THB1*	22.21	22.21
	10		22.16	
	10		22.26	
	10	*RACK1*	18.64	18.61
	10		18.59	
	10		18.60	
–S 6h dark	11	*THB1*	22.41	22.40
	11		22.42	
	11		22.38	
	11	*RACK1*	18.42	18.33
	11		18.30	
	11		18.27	

Table D. Comparison of relative THB1 expression in S-deprived *C. reinhardtii* cw15-325 cells incubated in the light or in the dark for 0.5h, 1h, 2h, 4h or 6h.

Variant	Average C_T *THB1*	Average C_T *RACK1*	ΔC_T	$\Delta\Delta C_T$	$2^{-\Delta\Delta CT}$
TAP	31.25±0.11	18.43±0.02	12.82		1
–S 0.5h light	22.76±0.07	18.80±0.02	3.95	-8.87	468.38
–S 1h light	22.50±0.12	18.83±0.03	3.67	-9.16	570.66
–S2h light	22.39±0.13	19.12±0.03	3.27	-9.55	751.08
–S 4h light	22.73±0.03	18.82±0.04	3.91	-8.91	481.49
–S 6h light	23.08±0.08	18.76±0.04	4.33	-8.50	360.92
–S 0.5h dark	22.19±0.08	18.51±0.01	3.68	-9.14	564.77
–S 1h dark	22.18±0.04	18.69±0.02	3.49	-9.33	644.69
–S2h dark	22.51±0.07	19.15±0.05	3.36	-9.46	704.56
–S 4h dark	22.21±0.05	18.61±0.03	3.60	-9.22	595.01
–S 6h dark	22.40±0.02	18.33±0.08	4.07	-8.75	431.09

Fig G. RNA integrity.

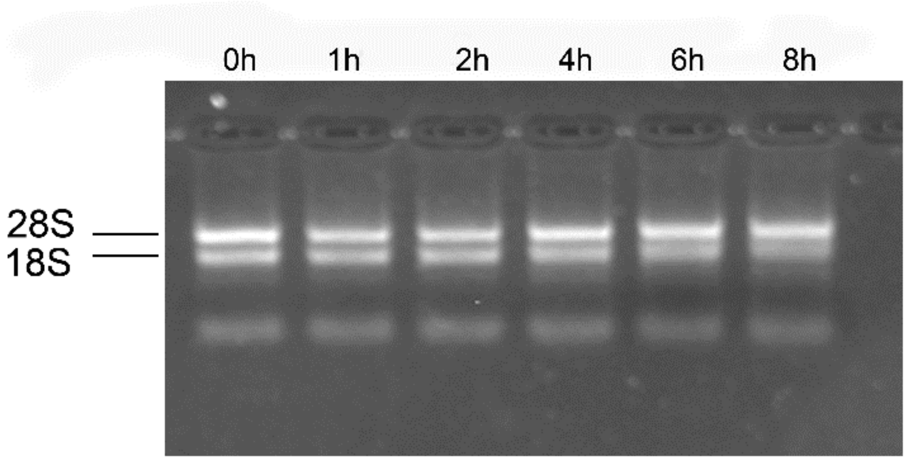

Fig H. Amplification chart of *ARS1*, *ARS2*, *SLT1*, *SLT2* and *SULTR2*, genes obtained from qRT-PCR analysis.

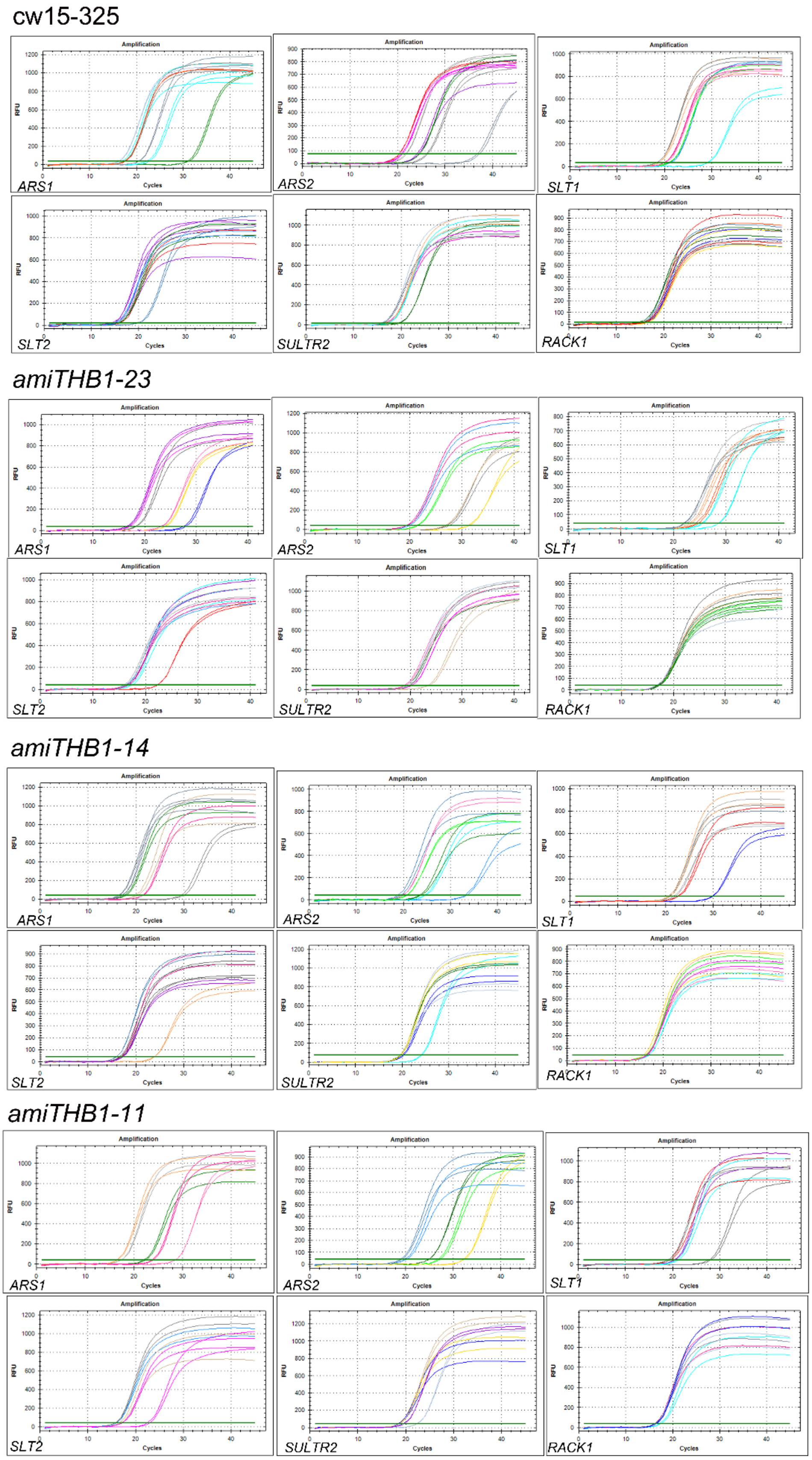

Table E. The Ct values for *ARS1, ARS2, SLT1, SLT2* and *SULTR2* genes across replicates in S-deprived *C. reinhardtii* cw15-325 and three *ami*THB1 strains incubated in the light for 1h, 2h, 4h, 6h or 8h.

Variant	Replicate	Gene	Theshold cycle (C_T)	C_T mean
**Cw15-325**				
TAP	1	*ARS1*	32.27	32.17
	1		32.04	
	1		32.21	
	1	*ARS2*	37.06	37.00
	1		36.94	
	1		37.01	
	1	*RACK1*	18.52	18.42
	1		18.39	
	1		18.36	
	1	*SLT1*	30.41	30.50
	1		30.56	
	1		30.52	
	1	*SLT2*	21.86	21.94
	1		22.03	
	1		21.92	
	1	*SULTR2*	21.49	21.46
	1		21.36	
	1		21.54	
	1	*RACK1*	18.59	18.47
	1		18.41	
	1		18.4	
–S 1h	2	*ARS1*	23.68	23.53
	2		23.44	
	2		23.48	
	2	*ARS2*	26.64	26.53
	2		26.41	
	2		26.53	
	2	*RACK1*	18.62	18.56
	2		18.56	
	2		18.50	
	2	*SLT1*	22.59	22.64
	2		22.71	
	2		22.62	
	2	*SLT2*	17.95	18.06
	2		18.21	
	2		18.02	

	2	*SULTR2*	19.71	19.84
	2		19.95	
	2		19.86	
	2	*RACK1*	18.71	18.59
	2		18.56	
	2		18.51	
–S 2h	3	*ARS1*	23.04	22.93
	3		22.85	
	3		22.90	
	3	*ARS2*	26.35	26.23
	3		26.13	
	3		26.21	
	3	*RACK1*	18.82	18.75
	3		18.67	
	3		18.76	
	3	*SLT1*	22.44	22.33
	3		22.25	
	3		22.29	
	3	*SLT2*	17.69	17.62
	3		17.51	
	3		17.66	
	3	*SULTR2*	19.86	19.77
	3		19.69	
	3		19.75	
	3	*RACK1*	18.92	18.81
	3		18.72	
	3		18.80	
–S 4h	4	*ARS1*	18.59	18.54
	4		18.47	
	4		18.57	
	4	*ARS2*	20.49	20.42
	4		20.34	
	4		20.42	
	4	*RACK1*	18.21	18.25
	4		18.34	
	4		18.20	
	4	*SLT1*	21.41	21.32
	4		21.24	
	4		21.31	
	4	*SLT2*	17.05	17.18
	4		17.27	
	4		17.21	
	4	*SULTR2*	19.35	19.28
	4		19.21	

	4		19.28	
	4	*RACK1*	18.21	18.27
	4		18.35	
	4		18.24	
-S 6h	5	*ARS1*	16.92	17.02
	5		17.11	
	5		17.02	
	5	*ARS2*	19.64	19.55
	5		19.43	
	5		19.58	
	5	*RACK1*	18.22	18.31
	5		18.39	
	5		18.31	
	5	*SLT1*	21.13	21.02
	5		20.97	
	5		20.95	
	5	*SLT2*	17.03	16.94
	5		16.82	
	5		16.96	
	5	*SULTR2*	19.18	19.08
	5		19.02	
	5		19.04	
	5	*RACK1*	18.44	18.34
	5		18.25	
	5		18.33	
–S 8h	6	*ARS1*	16.39	16.28
	6		16.21	
	6		16.24	
	6	*ARS2*	19.14	19.03
	6		18.93	
	6		19.01	
	6	*RACK1*	17.56	17.60
	6		17.72	
	6		17.52	
	6	*SLT1*	20.59	20.73
	6		20.84	
	6		20.75	
	6	*SLT2*	16.98	16.85
	6		16.75	
	6		16.82	
	6	*SULTR2*	17.86	17.93
	6		17.94	
	6		17.99	
	6	*RACK1*	17.69	17.62
	6		17.55	

	6		17.62	
amiTHB1-23				
TAP	1	*ARS1*	28.81	28.88
	1		28.95	
	1		28.88	
	1	*ARS2*	32.35	32.37
	1		32.45	
	1		32.32	
	1	*RACK1*	18.29	18.25
	1		18.27	
	1		18.19	
	1	*SLT1*	29.73	29.73
	1		29.64	
	1		29.81	
	1	*SLT2*	23.36	23.33
	1		23.28	
	1		23.35	
	1	*SULTR2*	24.67	24.60
	1		24.51	
	1		24.62	
	1	*RACK1*	18.11	18.21
	1		18.25	
	1		18.27	
–S 1h	2	*ARS1*	24.19	24.13
	2		24.09	
	2		24.12	
	2	*ARS2*	28.08	28.11
	2		28.19	
	2		28.06	
	2	*RACK1*	18.42	18.35
	2		18.29	
	2		18.33	
	2	*SLT1*	25.19	25.14
	2		25.09	
	2		25.14	
	2	*SLT2*	18.08	18.13
	2		18.21	
	2		18.09	
	2	*SULTR2*	20.26	20.21
	2		20.14	
	2		20.22	
	2	*RACK1*	18.39	18.33
	2		18.27	
	2		18.33	

–S 2h	3	*ARS1*	24.29	24.31
	3		24.39	
	3		24.25	
	3	*ARS2*	27.65	27.56
	3		27.50	
	3		27.54	
	3	*RACK1*	18.31	18.37
	3		18.42	
	3		18.37	
	3	*SLT1*	25.39	25.37
	3		25.27	
	3		25.45	
	3	*SLT2*	18.09	18.06
	3		17.96	
	3		18.13	
	3	*SULTR2*	21.14	21.20
	3		21.27	
	3		21.19	
	3	*RACK1*	18.31	18.36
	3		18.44	
	3		18.32	
–S 4h	4	*ARS1*	19.49	19.41
	4		19.32	
	4		19.43	
	4	*ARS2*	22.31	22.35
	4		22.48	
	4		22.26	
	4	*RACK1*	18.56	18.49
	4		18.41	
	4		18.49	
	4	*SLT1*	24.57	24.51
	4		24.45	
	4		24.53	
	4	*SLT2*	17.97	17.90
	4		17.89	
	4		17.84	
	4	*SULTR2*	20.79	20.94
	4		21.07	
	4		20.97	
	4	*RACK1*	18.50	18.45
	4		18.39	
	4		18.46	
-S 6h	5	*ARS1*	18.15	18.15
	5		18.21	
	5		18.09	

	5	*ARS2*	21.17	21.23
	5		21.34	
	5		21.19	
	5	*RACK1*	18.09	18.02
	5		17.89	
	5		18.07	
	5	*SLT1*	23.21	23.01
	5		22.89	
	5		22.94	
	5	*SLT2*	18.41	18.48
	5		18.56	
	5		18.47	
	5	*SULTR2*	20.64	20.72
	5		20.79	
	5		20.73	
	5	*RACK1*	17.89	17.99
	5		18.06	
	5		18.01	
–S 8h	6	*ARS1*	17.79	17.86
	6		17.95	
	6		17.83	
	6	*ARS2*	21.15	21.18
	6		21.26	
	6		21.13	
	6	*RACK1*	18.30	18.24
	6		18.19	
	6		18.22	
	6	*SLT1*	22.97	23.07
	6		23.14	
	6		23.10	
	6	*SLT2*	17.30	17.26
	6		17.15	
	6		17.34	
	6	*SULTR2*	20.04	20.13
	6		20.21	
	6		20.15	
	6	*RACK1*	18.24	18.25
	6		18.19	
	6		18.31	
amiTHB1-14				
TAP	1	*ARS1*	30.74	30.82
	1		30.94	
	1		30.78	
	1	*ARS2*	34.54	34.64
	1		34.78	

	1		34.61	
	1	*RACK1*	18.15	18.09
	1		18.04	
	1		18.07	
	1	*SLT1*	31.05	31.13
	1		31.20	
	1		31.13	
	1	*SLT2*	25.09	25.16
	1		25.29	
	1		25.11	
	1	*SULTR2*	24.41	24.46
	1		24.59	
	1		24.37	
	1	*RACK1*	18.02	18.12
	1		18.19	
	1		18.15	
–S 1h	2	*ARS1*	23.08	23.18
	2		23.24	
	2		23.21	
	2	*ARS2*	26.11	26.02
	2		25.89	
	2		26.05	
	2	*RACK1*	17.71	17.63
	2		17.54	
	2		17.64	
	2	*SLT1*	22.88	22.91
	2		22.99	
	2		22.85	
	2	*SLT2*	17.96	17.92
	2		17.84	
	2		17.95	
	2	*SULTR2*	19.87	19.86
	2		19.93	
	2		19.79	
	2	*RACK1*	17.56	17.68
	2		17.79	
	2		17.68	
–S 2h	3	*ARS1*	22.61	22.57
	3		22.40	
	3		22.69	
	3	*ARS2*	25.16	25.28
	3		25.37	
	3		25.31	
	3	*RACK1*	17.11	17.21

	3		17.25	
	3		17.26	
	3	*SLT1*	22.04	22.20
	3		22.31	
	3		22.25	
	3	*SLT2*	18.31	18.40
	3		18.50	
	3		18.39	
	3	*SULTR2*	19.89	19.90
	3		19.98	
	3		19.83	
	3	*RACK1*	17.21	17.25
	3		17.35	
	3		17.19	
–S 4h	4	*ARS1*	18.03	18.12
	4		18.22	
	4		18.11	
	4	*ARS2*	21.15	21.24
	4		21.36	
	4		21.20	
	4	*RACK1*	17.49	17.43
	4		17.35	
	4		17.45	
	4	*SLT1*	21.91	21.98
	4		22.06	
	4		21.97	
	4	*SLT2*	18.49	18.42
	4		18.34	
	4		18.42	
	4	*SULTR2*	20.12	20.02
	4		19.86	
	4		20.09	
	4	*RACK1*	17.39	17.48
	4		17.56	
	4		17.49	
-S 6h	5	*ARS1*	18.19	18.05
	5		17.94	
	5		18.01	
	5	*ARS2*	20.95	20.84
	5		20.76	
	5		20.81	
	5	*RACK1*	17.50	17.44
	5		17.32	
	5		17.49	

	5	*SLT1*	21.86	21.95
	5		22.03	
	5		21.95	
	5	*SLT2*	18.02	18.12
	5		18.25	
	5		18.08	
	5	*SULTR2*	20.51	20.60
	5		20.74	
	5		20.55	
	5	*RACK1*	17.41	17.48
	5		17.59	
	5		17.44	
–S 8h	6	*ARS1*	17.53	17.48
	6		17.40	
	6		17.51	
	6	*ARS2*	20.44	20.45
	6		20.35	
	6		20.56	
	6	*RACK1*	17.84	17.77
	6		17.74	
	6		17.72	
	6	*SLT1*	23.45	23.58
	6		23.69	
	6		23.60	
	6	*SLT2*	17.20	17.13
	6		17.04	
	6		17.15	
	6	*SULTR2*	19.40	19.48
	6		19.58	
	6		19.46	
	6	*RACK1*	17.89	17.81
	6		17.74	
	6		17.80	
amiTHB1-11				
TAP	1	*ARS1*	26.83	26.90
	1		26.95	
	1		26.92	
	1	*ARS2*	33.41	33.48
	1		33.56	
	1		33.46	
	1	*RACK1*	17.54	17.49
	1		17.41	
	1		17.51	
	1	*SLT1*	29.02	29.11

	1		29.19	
	1		29.11	
	1	*SLT2*	23.82	23.74
	1		23.69	
	1		23.70	
	1	*SULTR2*	23.46	23.37
	1		23.30	
	1		23.35	
	1	*RACK1*	17.5	17.44
	1		17.4	
	1		17.41	
–S 1h	2	*ARS1*	23.34	23.43
	2		23.51	
	2		23.43	
	2	*ARS2*	26.25	26.32
	2		26.41	
	2		26.30	
	2	*RACK1*	17.55	17.61
	2		17.69	
	2		17.59	
	2	*SLT1*	28.15	28.21
	2		28.29	
	2		28.20	
	2	*SLT2*	17.86	17.83
	2		17.74	
	2		17.90	
	2	*SULTR2*	19.60	19.65
	2		19.75	
	2		19.61	
	2	*RACK1*	17.64	17.56
	2		17.56	
	2		17.49	
–S 2h	3	*ARS1*	22.87	22.99
	3		23.05	
	3		23.06	
	3	*ARS2*	25.86	25.82
	3		25.81	
	3		25.79	
	3	*RACK1*	17.69	17.63
	3		17.55	
	3		17.65	
	3	*SLT1*	22.21	22.13
	3		22.06	
	3		22.11	
	3	*SLT2*	17.82	17.86

	3		17.95	
	3		17.82	
	3	*SULTR2*	20.49	20.44
	3		20.38	
	3		20.44	
	3	*RACK1*	17.65	17.57
	3		17.52	
	3		17.55	
–S 4h	4	*ARS1*	18.14	18.04
	4		17.95	
	4		18.04	
	4	*ARS2*	21.18	21.14
	4		21.05	
	4		21.19	
	4	*RACK1*	17.49	17.41
	4		17.33	
	4		17.41	
	4	*SLT1*	22.24	22.15
	4		22.09	
	4		22.12	
	4	*SLT2*	17.00	17.07
	4		17.12	
	4		17.08	
	4	*SULTR2*	20.59	20.67
	4		20.69	
	4		20.73	
	4	*RACK1*	17.31	17.37
	4		17.41	
	4		17.40	
-S 6h	5	*ARS1*	17.84	17.94
	5		18.02	
	5		17.95	
	5	*ARS2*	21.15	21.29
	5		21.39	
	5		21.32	
	5	*RACK1*	17.46	17.55
	5		17.68	
	5		17.50	
	5	*SLT1*	21.89	22.02
	5		22.11	
	5		22.06	
	5	*SLT2*	17.87	17.81
	5		17.74	
	5		17.83	
	5	*SULTR2*	20.60	20.65
	5		20.74	

	5		20.62	
	5	*RACK1*	17.44	17.52
	5		17.53	
	5		17.59	
	6	*ARS1*	17.61	17.56
	6		17.50	
	6		17.56	
	6	*ARS2*	20.69	20.65
	6		20.57	
	6		20.68	
	6	*RACK1*	17.44	17.52
	6		17.52	
	6		17.59	
–S 8h	6	*SLT1*	22.64	22.59
	6		22.52	
	6		22.61	
	6	*SLT2*	16.92	16.85
	6		16.85	
	6		16.79	
	6	*SULTR2*	19.29	19.31
	6		19.38	
	6		19.27	
	6	*RACK1*	17.54	17.49
	6		17.42	
	6		17.50	

Table F. Comparison of relative *ARS1, ARS2, SLT1, SLT2* and *SULTR2* expression in S-deprived *C. reinhardtii* cw15-325 and three *ami*THB1 strains incubated in the light for 1h, 2h, 4h, 6h or 8h.

**Cw15-325**				
Variant	Average C_T ***ARS1***	Average C_T *RACK1*	ΔC_T	$2^{-\Delta C_T}$
TAP	32.17±0.12	18.42±0.09	13.75	0.0000726
–S 1h	23.53±0.13	18.56±0.06	4.97	0.0319066
–S 2h	22.93±0.10	18.75±0.08	4.18	0.0551689
–S 4h	18.54±0.06	18.25±0.08	0.29	0.8179021
–S 6h	17.02±0.10	18.31±0.09	-1.29	2.4452806
–S 8h	16.28±0.10	17.60±0.10	-1.32	2.4966611

Variant	Average C_T ***ARS2***	Average C_T *RACK1*	ΔC_T	$2^{-\Delta C_T}$
TAP	37.00±0.06	18.42±0.09	18.58	0.0000026
–S 1h	26.53±0.12	18.56±0.06	7.97	0.0039883
–S 2h	26.23±0.11	18.75±0.08	7.48	0.0056014
–S 4h	20.42±0.08	18.25±0.08	2.17	0.2222107
–S 6h	19.55±0.11	18.31±0.09	1.24	0.4233727
–S 8h	19.03±0.11	17.60±0.10	1.43	0.3711309
Variant	Average C_T ***SLT1***	Average C_T *RACK1*	ΔC_T	$2^{-\Delta C_T}$
TAP	30.50±0.08	18.47±0.11	12.03	0.0002391
–S 1h	22.64±0.06	18.59±0.10	4.05	0.0603710
–S 2h	22.33±0.10	18.81±0.10	3.52	0.0871715
–S 4h	21.32±0.09	18.27±0.07	3.05	0.1207420
–S 6h	21.02±0.10	18.34±0.10	2.68	0.1560413
–S 8h	20.73±0.13	17.62±0.07	3.11	0.1158235
Variant	Average C_T ***SLT2***	Average C_T *RACK1*	ΔC_T	$2^{-\Delta C_T}$
TAP	21.94±0.09	18.47±0.11	3.47	0.0902456
–S 1h	18.06±0.13	18.59±0.10	-0.53	1.4439292
–S 2h	17.62±0.10	18.81±0.10	-1.19	2.2815274
–S 4h	17.18±0.11	18.27±0.07	-1.09	2.1287404
–S 6h	16.94±0.11	18.34±0.10	-1.40	2.6390158
–S 8h	16.85±0.12	17.62±0.07	-0.77	1.7052698
Variant	Average C_T ***SULTR2***	Average C_T *RACK1*	ΔC_T	$2^{-\Delta C_T}$
TAP	21.46±0.09	18.47±0.11	2.99	0.1258694
–S 1h	19.84±0.12	18.59±0.10	1.25	0.4204482
–S 2h	19.77±0.09	18.81±0.10	0.96	0.5140569
–S 4h	19.28±0.07	18.27±0.07	1.01	0.4965462

–S 6h	19.08±0.09	18.34±0.10	0.74	0.5987394
–S 8h	17.93±0.07	17.62±0.07	0.31	0.8066418
amiTHB1-23				
Variant	Average C_T ***ARS1***	Average C_T *RACK1*	ΔC_T	$2^{-\Delta C_T}$
TAP	28.88±0.07	18.25±0.05	10.63	0.0006310
–S 1h	24.13±0.05	18.35±0.07	5.78	0.0181990
–S 2h	24.31±0.07	18.37±0.06	5.94	0.0162885
–S 4h	19.41±0.09	18.49±0.08	0.92	0.5285090
–S 6h	18.15±0.06	18.02±0.11	0.13	0.9138315
–S 8h	17.86±0.08	18.24±0.06	-0.38	1.3013419
Variant	Average C_T ***ARS2***	Average C_T *RACK1*	ΔC_T	$2^{-\Delta C_T}$
TAP	32.37±0.07	18.25±0.05	14.12	0.0000562
–S 1h	28.11±0.07	18.35±0.07	9.76	0.0011533
–S 2h	27.56±0.08	18.37±0.06	9.19	0.0017121
–S 4h	22.35±0.12	18.49±0.08	3.86	0.0688691
–S 6h	21.23±0.09	18.02±0.11	3.21	0.1080672
–S 8h	21.18±0.07	18.24±0.06	2.94	0.1303082
Variant	Average C_T ***SLT1***	Average C_T *RACK1*	ΔC_T	$2^{-\Delta C_T}$
TAP	29.73±0.09	18.21±0.09	11.52	0.0003405
–S 1h	25.14±0.05	18.33±0.06	6.81	0.0089122
–S 2h	25.37±0.09	18.36±0.07	7.01	0.0077585
–S 4h	24.51±0.06	18.45±0.06	6.06	0.0149885
–S 6h	23.01±0.17	17.99±0.09	5.02	0.0308198
–S 8h	23.07±0.09	18.25±0.06	4.82	0.0354026
Variant	Average C_T ***SLT2***	Average C_T *RACK1*	ΔC_T	$2^{-\Delta C_T}$
TAP	23.33±0.04	18.21±0.09	5.12	0.0287559

–S 1h	18.13±0.07	18.33±0.06	-0.20	1.1486984
–S 2h	18.06±0.09	18.36±0.07	-0.30	1.2311444
–S 4h	17.90±0.07	18.45±0.06	-0.55	1.4640857
–S 6h	18.48±0.08	17.99±0.09	0.49	0.7120251
–S 8h	17.26±0.10	18.25±0.06	-0.99	1.9861850
Variant	Average C_T ***SULTR2***	Average C_T *RACK1*	ΔC_T	$2^{-\Delta C_T}$
TAP	24.60±0.08	18.21±0.09	6.39	0.0119239
–S 1h	20.21±0.06	18.33±0.06	1.88	0.2716837
–S 2h	21.20±0.07	18.36±0.07	2.84	0.1396609
–S 4h	20.94±0.14	18.45±0.06	2.49	0.1780063
–S 6h	20.72±0.08	17.99±0.09	2.73	0.1507260
–S 8h	20.13±0.09	18.25±0.06	1.88	0.2716837
***amiTHB1*-14**				
Variant	Average C_T ***ARS1***	Average C_T *RACK1*	ΔC_T	$2^{-\Delta C_T}$
TAP	30.82±0.11	18.09±0.06	12.73	0.0001472
–S 1h	23.18±0.09	17.63±0.09	5.55	0.0213444
–S 2h	22.57±0.15	17.21±0.08	5.36	0.0243489
–S 4h	18.12±0.10	17.43±0.07	0.69	0.6198538
–S 6h	18.05±0.13	17.44±0.10	0.61	0.6551967
–S 8h	17.48±0.07	17.77±0.06	-0.29	1.2226403
Variant	Average C_T ***ARS2***	Average C_T *RACK1*	ΔC_T	$2^{-\Delta C_T}$
TAP	34.64±0.12	18.09±0.06	16.55	0.0000104
–S 1h	26.02±0.11	17.63±0.09	8.39	0.0029810
–S 2h	25.28±0.11	17.21±0.08	8.07	0.0037212
–S 4h	21.24±0.11	17.43±0.07	3.81	0.0712977
–S 6h	20.84±0.10	17.44±0.10	3.40	0.0947323
–S 8h	20.45±0.11	17.77±0.06	2.68	0.1560413

Variant	Average C_T ***SLT1***	Average C_T *RACK1*	ΔC_T	$2^{-\Delta C_T}$
TAP	31.13±0.08	18.12±0.09	13.01	0.0001212
–S 1h	22.91±0.07	17.68±0.12	5.23	0.0266448
–S 2h	22.20±0.14	17.25±0.09	4.95	0.0323520
–S 4h	21.98±0.08	17.48±0.09	4.50	0.0441942
–S 6h	21.95±0.09	17.48±0.10	4.47	0.0451228
–S 8h	23.58±0.12	17.81±0.08	5.77	0.0183255
Variant	Average C_T ***SLT2***	Average C_T *RACK1*	ΔC_T	$2^{-\Delta C_T}$
TAP	25.16±0.11	18.12±0.09	7.04	0.0075989
–S 1h	17.92±0.07	17.68±0.12	0.24	0.8467453
–S 2h	18.40±0.10	17.25±0.09	1.15	0.4506252
–S 4h	18.42±0.08	17.48±0.09	0.94	0.5212329
–S 6h	18.12±0.12	17.48±0.10	0.64	0.6417129
–S 8h	17.13±0.08	17.81±0.08	-0.68	1.6021398
Variant	Average C_T ***SULTR2***	Average C_T *RACK1*	ΔC_T	$2^{-\Delta C_T}$
TAP	24.46±0.12	18.12±0.09	6.34	0.0123444
–S 1h	19.86±0.07	17.68±0.12	2.18	0.2206757
–S 2h	19.90±0.08	17.25±0.09	2.65	0.1593201
–S 4h	20.02±0.14	17.48±0.09	2.54	0.1719427
–S 6h	20.60±0.12	17.48±0.10	3.12	0.1150235
–S 8h	19.48±0.09	17.81±0.08	1.67	0.3142533
***amiTHB1*-11**				
Variant	Average C_T ***ARS1***	Average C_T *RACK1*	ΔC_T	$2^{-\Delta C_T}$
TAP	26.90±0.06	17.49±0.07	9.41	0.0014700
–S 1h	23.43±0.09	17.61±0.07	5.82	0.0177013
–S 2h	22.99±0.11	17.63±0.07	5.36	0.0243489

–S 4h	18.04±0.10	17.41±0.08	0.63	0.6461764
–S 6h	17.94±0.09	17.55±0.12	0.39	0.7631296
–S 8h	17.56±0.06	17.52±0.08	0.04	0.9726549
Variant	Average C_T ***ARS2***	Average C_T *RACK1*	ΔC_T	$2^{-\Delta C_T}$
TAP	33.48±0.08	17.49±0.07	15.99	0.0000154
–S 1h	26.32±0.08	17.61±0.07	8.71	0.0023880
–S 2h	25.82±0.04	17.63±0.07	8.19	0.0034242
–S 4h	21.14±0.08	17.41±0.08	3.73	0.0753630
–S 6h	21.29±0.12	17.55±0.12	3.74	0.0748424
–S 8h	20.65±0.07	17.52±0.08	3.13	0.1142289
Variant	Average C_T ***SLT1***	Average C_T *RACK1*	ΔC_T	$2^{-\Delta C_T}$
TAP	29.11±0.09	17.44±0.06	11.67	0.0003069
–S 1h	28.21±0.07	17.56±0.08	10.65	0.0006223
–S 2h	22.13±0.08	17.57±0.07	4.56	0.0423939
–S 4h	22.15±0.08	17.37±0.06	4.78	0.0363979
–S 6h	22.02±0.12	17.52±0.08	4.50	0.0441942
–S 8h	22.59±0.06	17.49±0.06	5.10	0.0291573
Variant	Average C_T ***SLT2***	Average C_T *RACK1*	ΔC_T	$2^{-\Delta C_T}$
TAP	23.74±0.07	17.44±0.06	6.30	0.0126914
–S 1h	17.83±0.08	17.56±0.08	0.27	0.8293195
–S 2h	17.86±0.08	17.57±0.07	0.29	0.8179021
–S 4h	17.07±0.06	17.37±0.06	-0.30	1.2311444
–S 6h	17.81±0.07	17.52±0.08	0.29	0.8179021
–S 8h	16.85±0.07	17.49±0.06	-0.64	1.5583292
Variant	Average C_T ***SULTR2***	Average C_T *RACK1*	ΔC_T	$2^{-\Delta C_T}$
TAP	23.37±0.08	17.44±0.06	5.93	0.0164018

–S 1h	19.65±0.08	17.56±0.08	2.09	0.2348807
–S 2h	20.44±0.06	17.57±0.07	2.87	0.1367867
–S 4h	20.67±0.07	17.37±0.06	3.30	0.1015315
–S 6h	20.65±0.08	17.52±0.08	3.13	0.1142289
–S 8h	19.31±0.06	17.49±0.06	1.82	0.2832210

Fig I. RNA integrity.

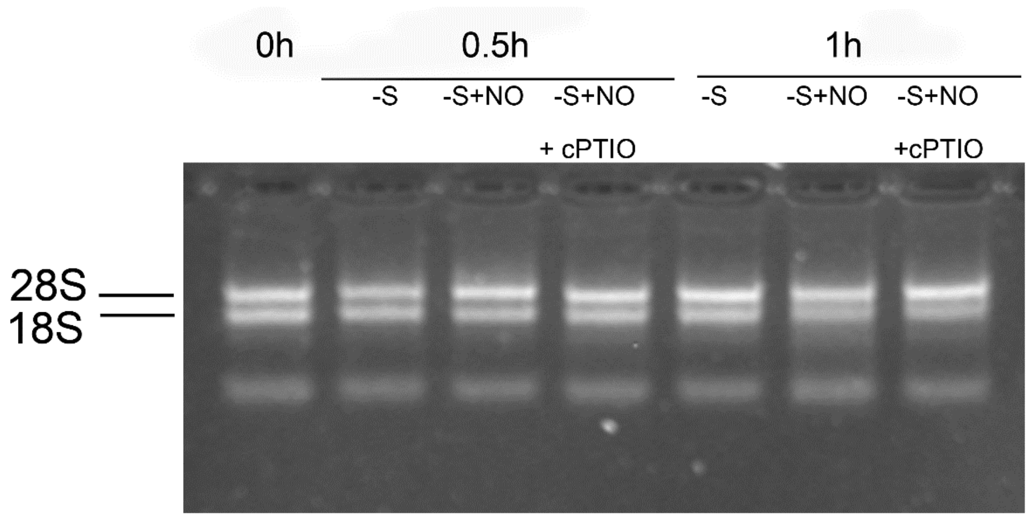

Fig J. Melt curve peaks of *ARS1*, *ARS2*, *SLT1*, *SLT2* and *SULTR2* genes obtained from qRT-PCR analysis.

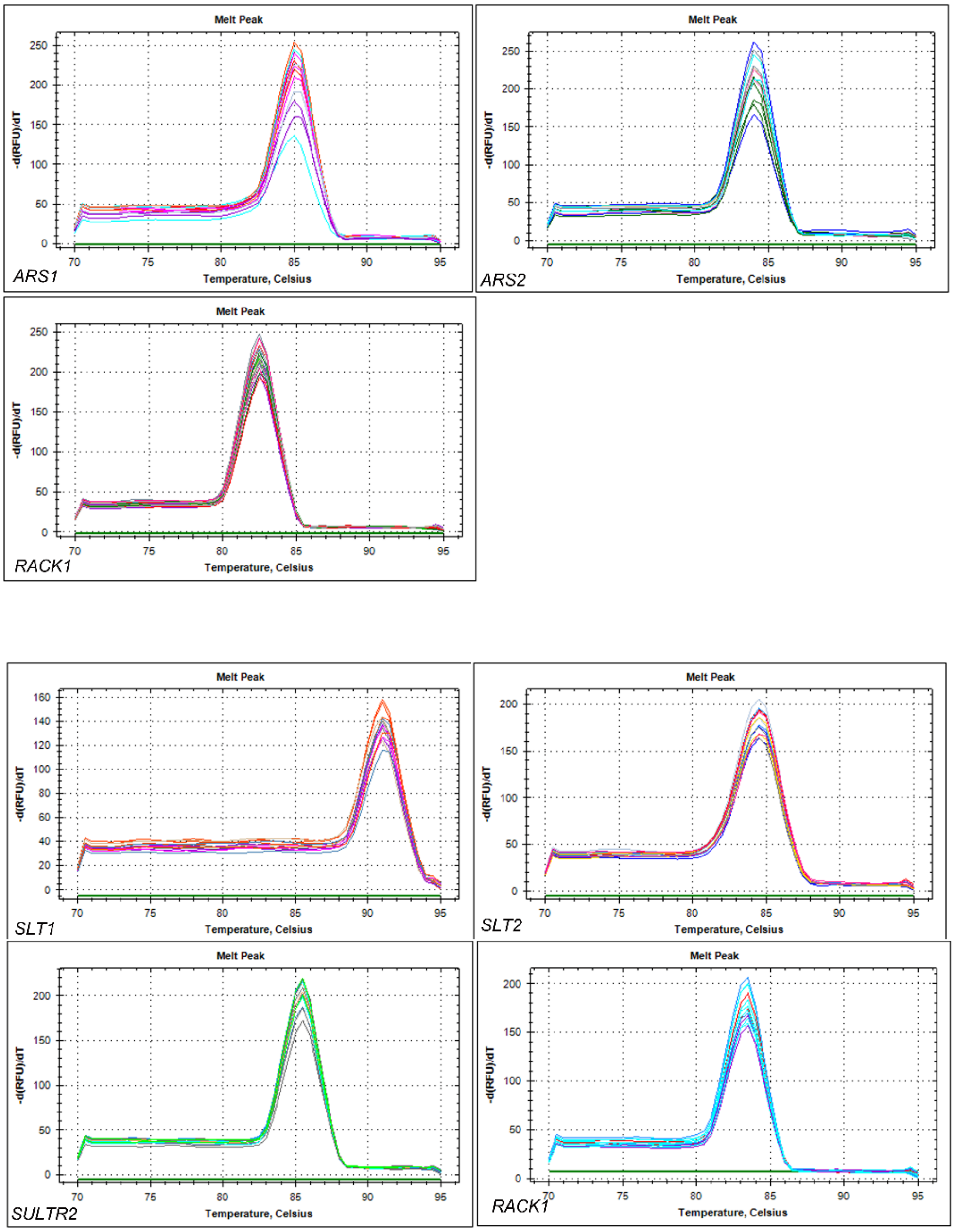

Fig K. Amplification chart of *ARS1*, *ARS2*, *SLT1*, *SLT2* and *SULTR2* genes obtained from qRT-PCR analysis.

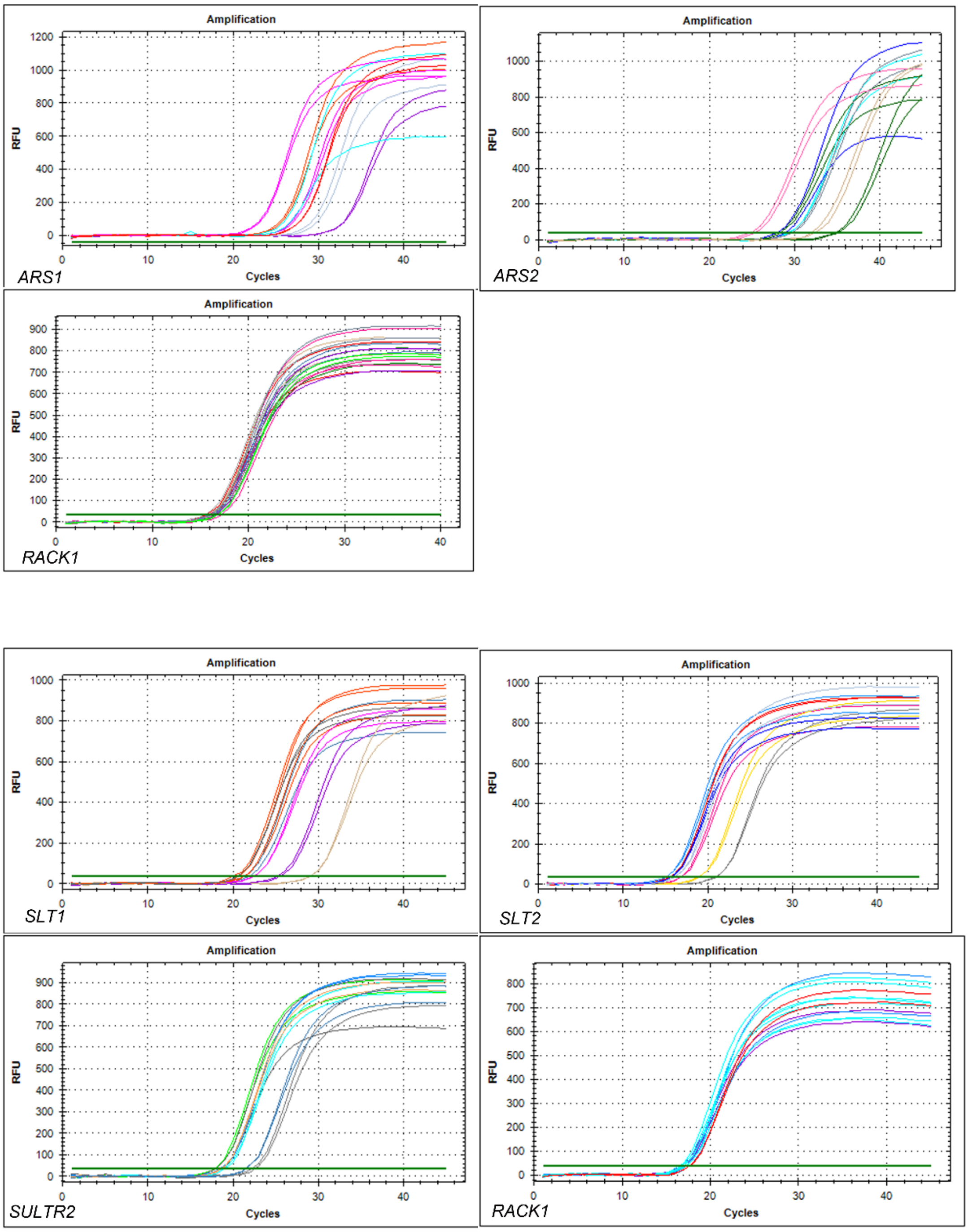

Table G. The Ct values for *ARS1, ARS2, SLT1, SLT2* and *SULTR2*genes across replicates in S-deprived *C. reinhardtii* cw15-325 cells incubated for 0.5 h or 1 h in the absence or presence of 50 µM DEA-NONOate (NO) with or without 100 µM cPTIO.

Variant	Replicate	Gene	Theshold cycle (Ct)	Ct mean
TAP	1	*ARS1*	33.21	33.17
	1		33.15	
	1		33.14	
	1	*ARS2*	36.53	36.34
	1		36.27	
	1		36.22	
	1	*RACK1*	17.45	17.47
	1		17.51	
	1		17.46	
	1	*SLT1*	30.50	30.48
	1		30.49	
	1		30.44	
	1	*SLT2*	22.35	22.31
	1		22.30	
	1		22.29	
	1	*SULTR2*	23.88	23.69
	1		23.61	
	1		23.57	
	1	*RACK1*	17.40	17.45
	1		17.47	
	1		17.47	
–S 0.5h	2	*ARS1*	25.90	26.01
	2		26.09	
	2		26.03	
	2	*ARS2*	30.06	30.11
	2		30.14	
	2		30.14	
	2	*RACK1*	17.46	17.41
	2		17.40	
	2		17.38	
	2	*SLT1*	21.91	21.97
	2		22.11	
	2		21.88	
	2	*SLT2*	16.46	16.42
	2		16.39	
	2		16.40	
	2	*SULTR2*	19.23	19.16
	2		19.14	
	2		19.12	

	2	*RACK1*	17.39	17.43
	2		17.45	
	2		17.44	
–S + NO 0.5h	3	*ARS1*	28.40	28.27
	3		28.19	
	3		28.21	
	3	*ARS2*	31.46	31.56
	3		31.72	
	3		31.49	
	3	*RACK1*	17.81	17.86
	3		17.92	
	3		17.84	
	3	*SLT1*	24.15	24.24
	3		24.34	
	3		24.23	
	3	*SLT2*	18.52	18.46
	3		18.39	
	3		18.46	
	3	*SULTR2*	20.79	20.69
	3		20.59	
	3		20.68	
	3	*RACK1*	17.85	17.82
	3		17.79	
	3		17.82	
-S + NO+cPTIO 0.5h	4	*ARS1*	25.25	25.19
	4		25.12	
	4		25.19	
	4	*ARS2*	28.41	28.28
	4		28.24	
	4		28.19	
	4	*RACK1*	17.25	17.28
	4		17.33	
	4		17.25	
	4	*SLT1*	20.91	20.95
	4		20.97	
	4		20.96	
	4	*SLT2*	16.01	16.05
	4		16.11	
	4		16.03	
	4	*SULTR2*	19.02	19.02
	4		19.10	
	4		18.95	
	4	*RACK1*	17.29	17.31
	4		17.34	
	4		17.31	

	5	*ARS1*	26.07	26.01
	5		26.03	
	5		25.93	
	5	*ARS2*	29.61	29.45
	5		29.33	
	5		29.42	
	5	*RACK1*	17.95	17.96
	5		17.98	
	5		17.94	
-S 1h	5	*SLT1*	23.08	22.97
	5		22.96	
	5		22.87	
	5	*SLT2*	17.23	17.28
	5		17.21	
	5		17.41	
	5	*SULTR2*	20.13	20.22
	5		20.28	
	5		20.24	
	5	*RACK1*	17.95	17.95
	5		17.97	
	5		17.93	
–S + NO 1h	6	*ARS1*	29.58	29.59
	6		29.49	
	6		29.69	
	6	*ARS2*	33.57	33.65
	6		33.79	
	6		33.59	
	6	*RACK1*	18.04	18.07
	6		18.06	
	6		18.12	
	6	*SLT1*	27.08	26.85
	6		26.79	
	6		26.69	
	6	*SLT2*	20.41	20.34
	6		20.31	
	6		20.30	
	6	*SULTR2*	23.01	22.93
	6		22.90	
	6		22.89	
	6	*RACK1*	18.08	18.03
	6		18.01	
	6		18.01	
–S + NO+cPTIO 1h	7	*ARS1*	23.28	23.19
	7		23.13	
	7		23.15	

	7	*ARS2*	26.03	26.01
	7		25.92	
	7		26.08	
	7	*RACK1*	18.29	18.27
	7		18.25	
	7		18.27	
	7	*SLT1*	22.46	22.37
	7		22.43	
	7		22.21	
	7	*SLT2*	17.21	17.36
	7		17.44	
	7		17.43	
	7	*SULTR2*	20.22	20.16
	7		20.04	
	7		20.21	
	7	*RACK1*	18.25	18.26
	7		18.29	
	7		18.23	

Table H. Comparison of relative *ARS1, ARS2, SLT1, SLT2* and *SULTR2* expression in S-deprived *C. reinhardtii* cw15-325 cells incubated for 0.5 h or 1 h in the absence or presence of 50 µM DEA-NONOate (NO) with or without 100 µM cPTIO.

Variant	Average C_T *ARS1*	Average C_T *RACK1*	ΔC_T	$\Delta\Delta C_T$	$2^{-\Delta\Delta C_T}$
TAP	33.17±0.04	17.47±0.03	15.70		1
–S 0.5h	26.01±0.10	17.41±0.04	8.60	-7.10	136.83
–S+NO 0.5h	28.27±0.12	17.86±0.06	10.41	-5.29	39.03
–S+NO+cPTIO 0.5h	25.19±0.07	17.28±0.05	7.91	-7.78	220.45
–S 1h	26.01±0.07	17.96±0.02	8.05	-7.65	200.39
–S+NO 1h	29.59±0.10	18.07±0.04	11.52	-4.18	18.13
–S+NO+cPTIO 1h	23.19±0.08	18.27±0.02	4.92	-10.78	1758.34

Variant	Average C_T *ARS2*	Average C_T *RACK1*	ΔC_T	$\Delta\Delta C_T$	$2^{-\Delta\Delta C_T}$
TAP	36.34±0.17	17.47±0.03	18.87		1
–S 0.5h	30.11±0.05	17.41±0.04	12.70	-6.17	71.98

–S+NO 0.5h	31.56±0.14	17.86±0.06	13.70	-5.17	36.00
–S+NO+cPTIO 0.5h	28.28±0.12	17.28±0.05	11.00	-7.87	233.56
–S 1h	29.45±0.14	17.96±0.02	11.49	-7.38	166.58
–S+NO 1h	33.65±0.12	18.07±0.04	15.58	-3.29	9.78
–S+NO+cPTIO 1h	26.01±0.08	18.27±0.02	7.74	-11.13	2241.11

Variant	Average C_T *SLT1*	Average C_T *RACK1*	ΔC_T	$\Delta\Delta C_T$	$2^{-\Delta\Delta C_T}$
TAP	30.48±0.03	17.45±0.04	13.03		1
–S 0.5h	21.97±0.13	17.43±0.03	4.54	-8.49	358.71
–S+NO 0.5h	24.24±0.10	17.82±0.03	6.42	-6.61	97.46
–S+NO+cPTIO 0.5h	20.95±0.03	17.31±0.03	3.64	-9.38	667.83
–S 1h	22.97±0.11	17.95±0.02	5.02	-8.01	257.40
–S+NO 1h	26.85±0.20	18.03±0.04	8.82	-4.20	18.42
–S+NO+cPTIO 1h	22.37±0.14	18.26±0.03	4.12	-8.91	479.93

Variant	Average C_T *SLT2*	Average C_T *RACK1*	ΔC_T	$\Delta\Delta C_T$	$2^{-\Delta\Delta C_T}$
TAP	22.31±0.03	17.45±0.04	4.86		1
–S 0.5h	16.42±0.04	17.43±0.03	-1.01	-5.88	58.76
–S+NO 0.5h	18.46±0.07	17.82±0.03	0.64	-4.22	18.68
–S+NO+cPTIO 0.5h	16.05±0.05	17.31±0.03	-1.26	-6.12	69.71
–S 1h	17.28±0.11	17.95±0.02	-0.67	-5.53	46.25
–S+NO 1h	20.34±0.06	18.03±0.04	2.31	-2.55	5.87
–S+NO+cPTIO 1h	17.36±0.13	18.26±0.03	-0.89	-5.75	53.94

Variant	Average C_T *SULTR2*	Average C_T *RACK1*	ΔC_T	$\Delta\Delta C_T$	$2^{-\Delta\Delta C_T}$
TAP	23.69±0.17	17.45±0.04	6.24		1

–S 0.5h	19.16±0.06	17.43±0.03	1.73	-4.50	22.68
–S+NO 0.5h	20.69±0.10	17.82±0.03	2.87	-3.37	10.32
–S+NO+cPTIO 0.5h	19.02±0.08	17.31±0.03	1.71	-4.53	23.05
–S 1h	20.22±0.08	17.95±0.02	2.27	-3.97	15.61
–S+NO 1h	22.93±0.07	18.03±0.02	4.90	-1.33	2.52
–S+NO+cPTIO 1h	20.16±0.10	18.26±0.03	1.91	-4.33	20.07
