# Supplementary material for: Truncated hemoglobin 1 is a new player in Chlamydomonas reinhardtii acclimation to sulfur deprivation
Source: PLoS One. 2017 Oct 19;12(10):e0186851. doi: 10.1371/journal.pone.0186851 (PMC5648252; doi:10.1371/journal.pone.0186851)
Supplement: S1 Table — (DOCX) [file pone.0186851.s005.docx]

**S1 Table. Primer list**

| Target gene/Accession number | Primer name | Sequence (5´–3´) | Reference |
| --- | --- | --- | --- |
| *THB1/*Cre14.g615400 | *THB1*F  *THB1*R | ATGAAGAAGCAGCGCCGCAAAC ACCAGGTCAAAGTGGTGGTGGTTC | [18] |
| *THB2/*Cre14.g615350 | *THB2*F  *THB2*R | GCCGGTTGATCCGCGACAAG CGATCCAACTTTTACACCCGCTCAA | [18] |
| *THB3/*Cre04.g218800 | *THB3*F *THB3*R | TCACTTATCGCCAGTCTAGAGGAC CGCTCAGGATGTCGTCTATAAGC | [18] |
| *THB4/*Cre04.g218750 | *THB4*F  *THB4*R | GCTTCAAGGAGACGGTGTGAAGTCTAC ACATCCACCGTTGCTGCCACA | [18] |
| *THB5/*Cre07.g351100 | *THB5*F  *THB5*R | GGCGTTTTATCGCAAGTTGT  CTTGAACGTATCCAGCAGCA | This work |
| *THB6/*Cre16.g654250 | *THB6*F  *THB6*R | CCTGGACTCGATAGCAGAGG  TGTCGTGAGAGACGGAACTG | This work |
| *THB7/*Cre16.g661000 | *THB7*F  *THB7*R | CATGGTGCCGTGCTCGTACA  CGACCAGCACTGCCTACTTG | [15] |
| *THB8/*Cre16.g661200 | *THB8*F  *THB8*R | CGGGAGTCAGCAAGCTGTCAAC  CCGCCCGTACACAAACAAGCAC | [15] |
| *THB9/*Cre16.g661250 | *THB9*F  *THB9*R | GCTCTCTCTGGTTTTGAAGCAT  AGCTGCTCATCTGCGTACAAT | This work |
| *THB10/*Cre16.g661300 | *THB10*F  *THB10*R | TGCTGCGGAGGTGTTCCTTG CATTGCCGCCTCTGCTGATG | [15] |
| *THB11/*Cre16.g662750 | *THB11*F  *THB11*R | TTGCGTGCGTCCATGCTGTC  CCGGTTGCGGATACACCTCT | [15] |
| *THB12/*Cre16.g663000 | *THB12*F  *THB12*R | GACCCCTCACTCATAAAGTTCCT  AAGTACTTCATGCCCAGATCAAA | This work |
| *RACK1/*Cre06.g278222 | *RACK1*F  *RACK1*R | CTTCTCGCCCATGACCAC  CCCACCAGGTTGTTCTTCAG | Zalutskaya et al., 2015. |
| *ARS1/*Cre16.g671400 | *ARS1*F  *ARS1*R | CGCGCCGTCACTTGTTTGTTG  GCCCACTTCTTTACCCAGC | This work |
| *ARS2/*Cre16.g671350 | *ARS2*F  *ARS2*R | CTTAATTGCATGCGCGCCGTCA  TCAGAACACCAACGCAAGTTTCCAG | [28] |
| *SULTR2/*Cre17.g723350 | *SULTR2*F  *SULTR2*R | ACGTGGCATGCAGCTCAT  CTTGCCACTTTGCCAGGT | [28] |
| *SLT1/*Cre12.g502600 | *SLT1*F  *SLT1*R | ACGGGTCTTCGAGCGAATTGC  CGACTGCTTACGCAACAATCTTGG | [28] |
| *SLT2/*Cre10.g445000 | *SLT2*F  *SLT2*R | TGTCGCGATATCGTTCATCA  TGCGACAAAGTCTGCCAAGT | Fang et al., 2014. |

Sequences were obtained from Phytozome 12, *Chlamydomonas reinhardtii* *v5.5*

Fang S-C, Chung C-L, Chen C-H, Lopez-Paz C, Umen J. Defects in a new class of sulfate/anion transporter link sulfur acclimation responses to intracellular glutathione levels and cell cycle control. Plant Physiol. 2014; 166: 1852–1868.

Zalutskaya Zh, Lapina T, Ermilova E. The *Chlamydomonas reinhardtii* alternative oxidase 1 is regulated by heat stress. Plant Physiol. Biochem. 2015; 97: 229–234.
